# The Two Faces of the Liquid Ordered Phase

**DOI:** 10.1021/acs.jpclett.1c03712

**Published:** 2022-02-01

**Authors:** Itay Schachter, Riku O. Paananen, Balázs Fábián, Piotr Jurkiewicz, Matti Javanainen

**Affiliations:** † Institute of Organic Chemistry and Biochemistry of the Czech Academy of Sciences, Flemingovo nám. 542/2, CZ-16000 Prague 6, Czech Republic; ‡ Institute of Chemistry, the Fritz Haber Research Center, and the Harvey M. Kruger Center for Nanoscience & Nanotechnology, The Hebrew University, Jerusalem 9190401, Israel; § Department of Chemistry, FI-00014 University of Helsinki, Helsinki, Finland; ∥ Department of Ophthalmology, FI-00014 University of Helsinki and Helsinki University Hospital, Helsinki, Finland; ⊥ J. Heyrovský Institute of Physical Chemistry of the Czech Academy of Sciences, Dolejškova 2155/3, CZ-18223 Prague 8, Czech Republic; # Institute of Biotechnology, FI-00014 University of Helsinki, Helsinki, Finland

## Abstract

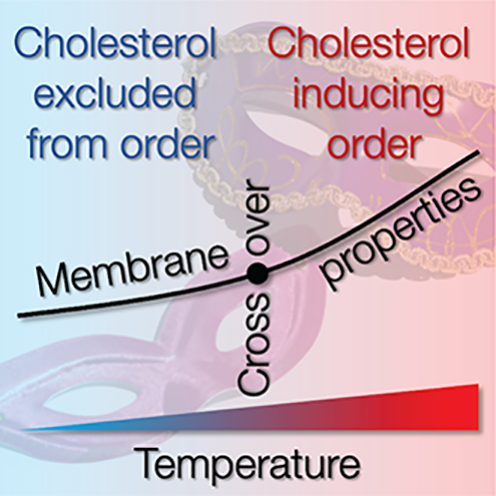

Coexisting liquid ordered (L_o_) and liquid disordered (L_d_) lipid phases in synthetic and plasma membrane-derived vesicles are commonly used to model the heterogeneity of biological membranes, including their putative ordered rafts. However, raft-associated proteins exclusively partition to the L_d_ and not the L_o_ phase in these model systems. We believe that the difference stems from the different microscopic structures of the lipid rafts at physiological temperature and the L_o_ phase studied at room temperature. To probe this structural diversity across temperatures, we performed atomistic molecular dynamics simulations, differential scanning calorimetry, and fluorescence spectroscopy on L_o_ phase membranes. Our results suggest that raft-associated proteins are excluded from the L_o_ phase at room temperature due to the presence of a stiff, hexagonally packed lipid structure. This structure melts upon heating, which could lead to the preferential solvation of proteins by order-preferring lipids. This structural transition is manifested as a subtle crossover in membrane properties; yet, both temperature regimes still fulfill the definition of the L_o_ phase. We postulate that in the compositionally complex plasma membrane and in vesicles derived therefrom, both molecular structures can be present depending on the local lipid composition. These structural differences must be taken into account when using synthetic or plasma membrane-derived vesicles as a model for cellular membrane heterogeneity below the physiological temperature.

When inserted into certain phospholipid membranes in sufficient amounts, cholesterol (CHOL) induces the liquid ordered (L_o_) phase,^1^ which is an intermediate between the fluid liquid disordered (L_d_) and solid (gel) phases. As the name suggests, the L_o_ phase displays gel-like ordering of the lipid acyl chains, whereas the lipid rotational and translational dynamics are relatively fast akin to the L_d_ phase, highlighting the fluid character of the membrane. The addition of CHOL increases the order, thickness, and stiffness of the L_d_ phase, yet has the opposite effects on the gel phase.^2^ As a result, CHOL renders these two phases more similar. Binary mixtures of CHOL and certain phospholipids have a threshold CHOL concentration, above which the first-order L_d_–gel transition at *T*_m_ vanishes. For dipalmitoylphosphatidylcholine (DPPC), this happens at ∼25–30 mol % of CHOL, above which many membrane properties show continuous temperature dependence, indicating a uniform L_o_ phase. This behavior is reported by numerous experimental approaches^3−8^ and simulations.^9−12^ Still, some studies have reported nonidealities within the L_o_ phase: differential scanning calorimetry (DSC) and NMR detected a broad transition,^13,14^ which was suggested to correspond to the transition between two distinct L_o_ phases differing in the lipid chain tilt.^14,15^ NMR spectroscopy and X-ray scattering suggested that even at high CHOL concentrations, heating modifies the lipid structure at the glycerol level, repositions CHOL toward lipid headgroups, and disorders the acyl chain termini.^16,17^

Certain ternary mixtures of CHOL with low-*T*_m_ and high-*T*_m_ lipids display L_o_/L_d_ phase coexistence^18^ and are commonly used to model the putative nanoscale ordered lipid domains (“rafts”) in biomembranes.^19,20^ Notably, this coexistence is detected in model systems only at temperatures below the *T*_m_ of the high-*T*_m_ lipid. Also, giant plasma membrane-derived vesicles (GPMVs) phase-separate well below the body temperature.^21^ This agrees with the ability of CHOL to break the gel phase to an L_o_ one. Indeed, recent simulations have demonstrated that the L_o_ phase has an internal structure containing small hexagonally packed and CHOL-depleted regions,^22−24^ despite CHOL’s preference for saturated lipid chains in fluid phases.^25^ Experiments have struggled to provide a consistent molecular view of the L_o_ phase in binary mixtures, and the situation is at least equally complicated for ternary mixtures: NMR^26^ and X-ray scattering^27^ resolve signals originating from the two coexisting phases; yet, their compositions are temperature-dependent. Importantly, it is unclear whether the L_o_ phase observed in phase-separated vesicles differs structurally from that observed for the same mixture at physiological *T* and whether the former is a faithful model for plasma cell membrane heterogeneity.^28^ Surprisingly, raft-associated proteins partition to the L_d_ phase in phase-separated synthetic vesicles,^29^ although the same proteins can locate to the ordered phase in phase-separated GMPVs.^30^ Moreover, there are even differences between the partitioning behavior of proteins in GMPVs^31^ and plasma membrane spheres^32^ since the latter can maintain phase separation at higher temperatures due to mechanisms not present in model systems.^32^ Still, very few transmembrane domains are targeted to the L_o_ phase even in phase-separated GMPVs.^31,33^ In general, the two coexisting phases in different model systems and *in vivo* have different levels of mutual similarity.^34−37^ These findings indeed suggest that the putative rafts likely differ from the L_o_ phase observed in synthetic vesicles more than only by their size,^21,28^ and some of these factors might also be temperature-dependent.^38^

To shed light on the structure of ordered lipid phases across temperatures, we performed differential scanning calorimetry (DSC) and fluorescence measurements of binary and ternary L_o_ lipid mixtures, and provided a molecular picture of the observed phenomena using atomistic molecular dynamics simulations. We studied bilayers consisting of (1) a *ternary mixture* of 55 mol % DPPC, 15 mol % DOPC, and 30 mol % CHOL, and (2) a *binary* mixture of 70 mol % DPPC and 30 mol % CHOL. The composition of the *ternary* mixture reflects the L_o_ phase of a phase-separated 0.40/0.40/0.20 (DPPC/DOPC/CHOL) lipid bilayer at 298 K.^22,39^ On the basis of the phase diagrams, both mixtures remain in the L_o_ phase at all studied temperatures, ranging from 293 K to 333 K.^8,39,40^

Our simulations revealed distinct changes in the bilayer structure depending on the temperature, as shown in Figure 1 (see Figures S1 and S2 for more temperatures). Both in *binary* and *ternary* mixtures, DPPC chains mainly adapt an *anti* conformation at 293 K; yet, the amount of *gauche* isomers is significantly increased at 333 K. However, at 313 K the two mixtures differ as DPPC lipids remain substantially ordered in the *binary* mixture. This indicates that DPPC undergoes some kind of a transition, yet at different temperatures for the two mixtures. The question is whether this transition is manifested in membrane properties.

**Figure 1:**
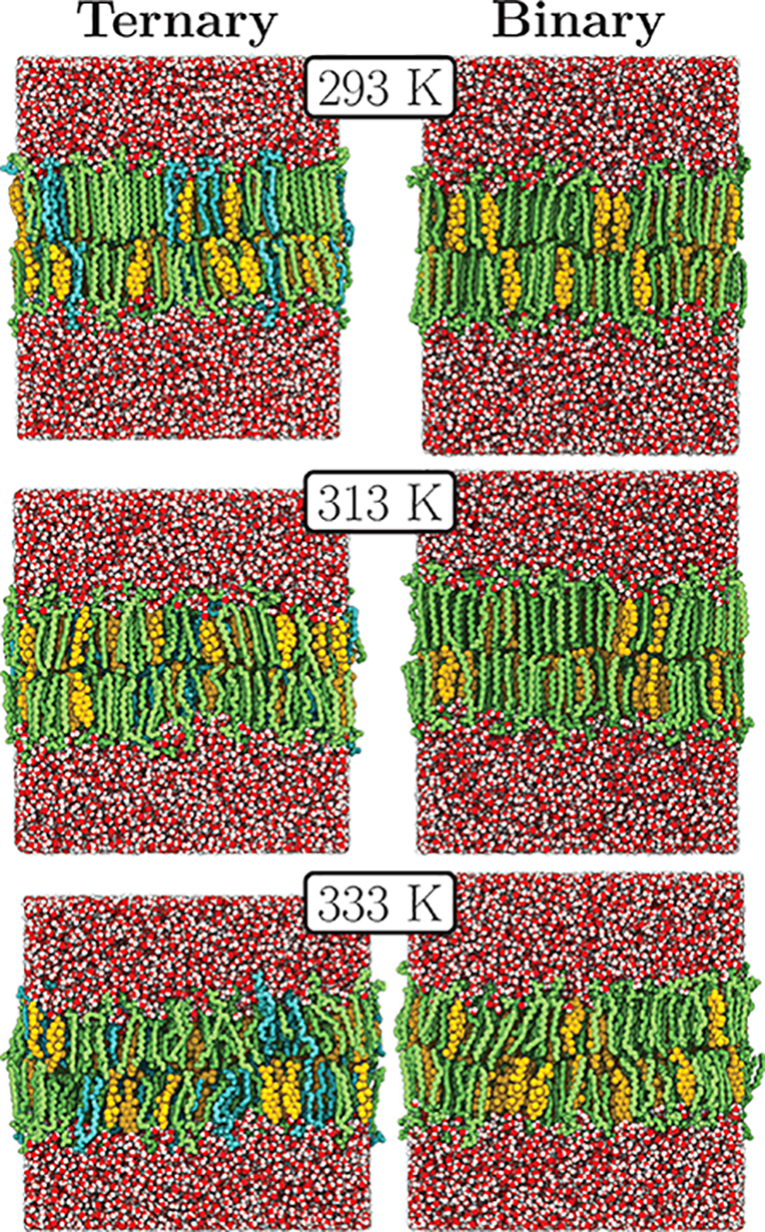
Final structures (after 1 μs of simulation) of *ternary* (left) and *binary* (right) mixtures at 293 K (top), 313 K (middle), and 333 K (bottom). The chains are mainly in an *anti* conformation at 293 K; yet, they melt at 333 K, resulting in an increase in the amount of chains in the *gauche* conformation. Here, DPPC is shown in green, DOPC in blue, and CHOL in yellow. Lipid hydrogens and ions are omitted. Water is shown in red and white.

As demonstrated in Figure 2, the answer is affirmative. The area per phospholipid (APPL, shown in Figure 2A) exhibits two regimes with different thermal expansion coefficients and a crossover at *T*_co_^ter^ ≈ 308 K (*ternary* mixture) or *T*_co_^bin^ ≈ 318 K (*binary* mixture). The coefficients differ between the *binary* and *ternary* mixtures in both temperature regimes. The *ternary* mixture contains fluid DOPC, which explains the slightly larger coefficient below *T*_co_.^41^

**Figure 2:**
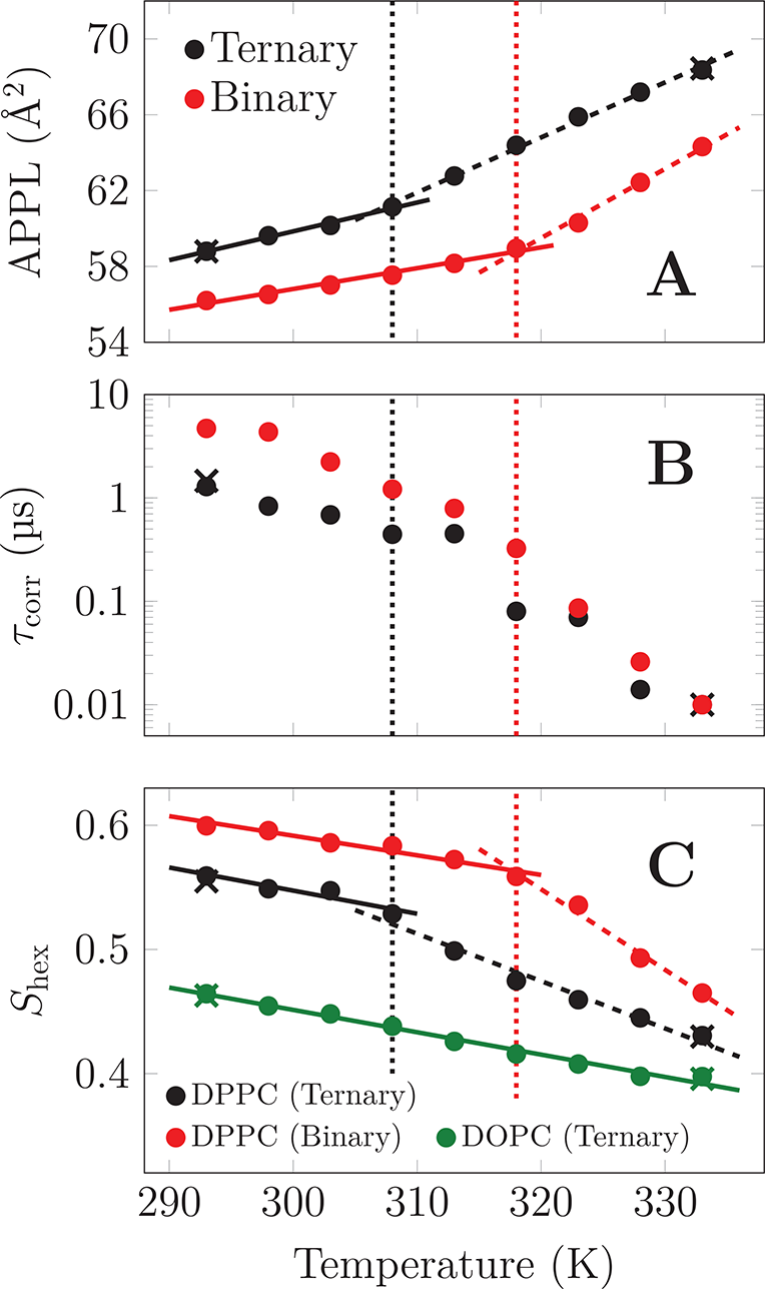
Temperature dependence of membrane properties for the *binary* and *ternary* mixtures. The crosses show data extracted from additional simulations that were performed to ensure that hysteresis did not affect the results (see Methods in the Supporting Information). (A) Area per phospholipid (APPL) shows two different slopes, i.e., two different thermal expansion coefficients (solid and dashed lines) with a crossover at either *T*_co_^ter^ ≈ 308 K or *T*_co_^bin^ ≈ 318 K, highlighted by the vertical dotted lines. (B) The correlation time of the autocorrelation function of the orientation of the glycerol backbone. The autocorrelation data are shown in Figure S3. (C) The hexatic order parameter of the lipid chains. The parameter values range from 0 to 1. No change of slope in the *ternary* system is observed for DOPC.

DSC is the tool of choice to detect phase transitions or more subtle molecular rearrangements. We performed such measurements on large unilamellar vesicles for the binary and ternary mixtures (see Methods in the Supporting Information). As shown in Figure 4A, DSC detects broad peaks for the *binary* and *ternary* systems, and their maxima agree perfectly with the crossover temperatures found in our simulations (see labels in Figure 4A). The absence of a first-order phase transition suggests that a pure L_o_ phase is present at all temperatures. Nonetheless, it undergoes a minor structural transition. Measurements of pure DPPC reveal a well-defined sharp gel–liquid transition at *T*_m_ = 314 K, whereas binary and ternary mixtures with only 15% CHOL display L_o_/gel coexistence at low *T*. This results in the presence of both a sharp and a broad peak (Figure S10), which are associated with the melting of CHOL-poor gel and CHOL-rich L_o_ phases.^13,15^

We then verified whether lipids really exhibit L_o_-like fluidity and order both below and above *T*_co_. The characteristic rotational times of the DPPC glycerol backbone in the *binary* and *ternary* systems are shown in Figure 2B. The values are indeed considerably smaller than those of the gel phase.^42^ The rotational times decrease continuously upon heating in the studied temperature range by two orders of magnitude both for *binary* and *ternary* mixtures. Our values agree reasonably well with those measured for the binary mixture at 285 K.^43^ The fluid character of the lipid bilayer can also be concluded by comparing lateral diffusion coefficients of lipids, shown in Figure S4, with experimental data in different phases.^3^ Even though the membranes can be considered fluid across all studied temperatures, the dynamics still speed up significantly above *T*_co_. Additionally, we verified that the mean deuterium order parameters show high L_o_-like ordering at all studied temperatures (see Figure S5).

Next, we looked into lipid packing in the bilayers in detail. We used the in-plane hexatic order parameter *S*_hex_ of chosen atoms in the acyl chain region to characterize the degree of hexagonal arrangement of lipid molecules.^22−24^ As shown in Figure 2C, DPPC shows relatively high *S*_hex_ values for both the *binary* and *ternary* mixtures below *T*_co_. However, after the crossover they converge rapidly toward values extracted for DOPC in the *ternary* mixture. The *S*_hex_ of DPPC is slightly higher in the *binary* mixture due to the lack of packing perturbations by DOPC. The absence of any crossovers in the DOPC curve indicates that this lipid is excluded from the hexagonally packed clusters. These findings agree with our movies showing the melting of the clusters in the *ternary* mixture at *T*_co_^ter^, available at DOI: 10.6084/m9.figshare.13176167. Lipid exchange rates in these clusters, shown in Figure S6, also show a crossover at *T*_co_.

We next evaluated whether structural changes, suggested previously to take place within the L_o_ phase,^13−17^ are present in our simulations. As shown in Figure 3A, the mean tilt angle of DPPC acyl chains shows two different slopes with crossovers at *T*_co_, in line with the predicted variation in chain tilting within the L_o_ region.^15^ Clarke et al. and Reinl et al. suggested that the temperature increase within the L_o_ phase shifts CHOL toward the headgroup region, leading to the disordering of the lipid chain terminal carbons.^16,17^ This repositioning of CHOL toward the headgroup region indeed takes place in our simulations (Figure S7). This process further decreases the acyl chain ordering in the membrane core. Indeed, as shown in Figure 3B, the mean deuterium order parameter of the last nine carbons of the DPPC sn-2 chain displays a clear decrease with crossovers at *T*_co_. The glycerol region melting, suggested to occur within the L_o_ phase by Clarke et al.,^16^ also takes place as indicated by the significant increase in glycerol dynamics in Figure 2B, albeit with a less pronounced crossover.

DPPC chains can be categorized based on their local structure. Our algorithm (see Methods in the Supporting Information) distinguishes “core” chains encapsulated in the tightly-packed clusters, “edge” chains mainly at cluster edges, and “free” chains that are excluded from the clusters (see Figure S8 for demonstration). The mean deuterium order parameter , shown in Figure 3C, demonstrates that “core” chains are the most ordered at all temperatures. Notably, they are also the furthest (0.90 nm on average) away from CHOL molecules. The “free” chains are the least ordered and closest to a CHOL (0.62 nm), whereas “edge” chains show intermediate behavior. This agrees with the exclusion of CHOL from the tightly packed clusters in ref (22). As shown in Figure S9, the “core” chains are also the least tilted, followed by the “edge” chains, and finally the “free” chains. The temperature dependencies of both  and chain tilt consist of two factors, as demonstrated in Figures 3C and S9: The “core”, “edge”, and “free” chain populations depend on the temperature with the “core” fraction declining rapidly after *T*_co_, corresponding to the melting of the clusters. Moreover, while “free” chains show a continuous change in ordering and tilt, there is a crossover in the values of the “core” and “edge” chains at *T*_co_. These two factors contribute to the striking change of slope in the temperature dependencies of these properties in Figure 3.

**Figure 3:**
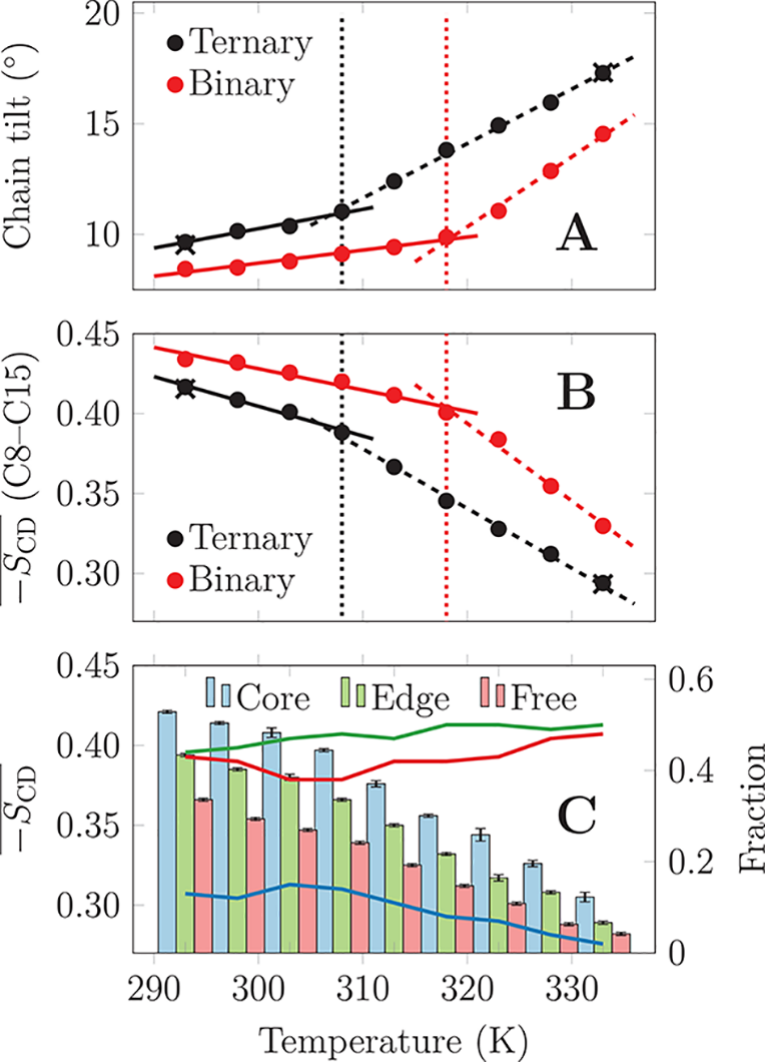
Membrane properties which were suggested to differ across temperature in the L_o_ regime by earlier experimental studies. The crosses correspond to additional simulations that demonstrate that hysteresis does not affect the obtained results (see Methods in the Supporting Information). (A) DPPC chain tilt shows a change in slope at the crossover temperatures of *T*_co_^ter^ ≈ 308 K and *T*_co_^bin^ ≈ 318 K. (B) The mean order parameter of all DPPC sn-2 chains, starting from the eighth carbon, i.e., the middle of the chain. The carbon-wise order parameter plots are shown in Figure S5. (C) Mean order parameter of DPPC chains as a function of cluster identity (bars) and the fractions of the different cluster identities (lines). “Core” chains are surrounded by hexagonally packed chains, “free” chains are not part of a hexagonally packed cluster, and “edge” points roughly correspond to the cluster edges.

To provide further experimental evidence for the described changes in the molecular structure, we performed two complementary fluorescence experiments on large unilamellar vesicles. First, we utilized Patman—a fluorescent polarity probe stably located at the lipid carbonyl region of all membrane phases.^44,45^ Upon electronic excitation, a charge transfer over the naphthalene ring provides a major change in the dipole moment of Patman. Subsequently, its Stokes shift provides information on the hydration and mobility of the carbonyl region—both highly sensitive to the lipid phase state.^46^ Patman generalized polarization (GP) was calculated based on the probe emission at 420 nm (typical for gel and L_o_ phases) and 495 nm (characteristic for L_d_). Thus, GP contains information on both lipid fluidity and hydration. High GP values associated with gel and L_o_ phases, and low ones with the L_d_ phase. For details, see the Methods in the Supporting Information.

GP results of the *binary* and *ternary* mixtures, as well as DPPC (gel–L_d_*T*_m_ = 314 K) and POPC (L_d_ across all measured temperatures), are shown in Figure 4B. In all systems, the emitted wavelengths red-shift gradually when the temperature increases, reflecting an increased mobility of the lipid carbonyls (Figure S11). For POPC, GP reflects increasing lipid mobility in the L_d_ phase with increasing temperature. This change is continuous and saturates at higher temperatures. On the other hand, GP measured for DPPC displays an abrupt jump at *T*_m_ as the lipid mobility changes drastically. Please note that below and above *T*_m_, GP is still decreasing with increasing temperature. The GP curves for the *binary* and *ternary* mixtures present character intermediate to that of POPC and DPPC. The changes are much smoother than for DPPC, but the fits by modified Boltzmann sigmoidal functions (see the Supporting Information) still display clear inflection points that correspond to the *T*_co_ extracted from our simulations and DSC (see Table S3 for all the fitting parameters).

**Figure 4:**
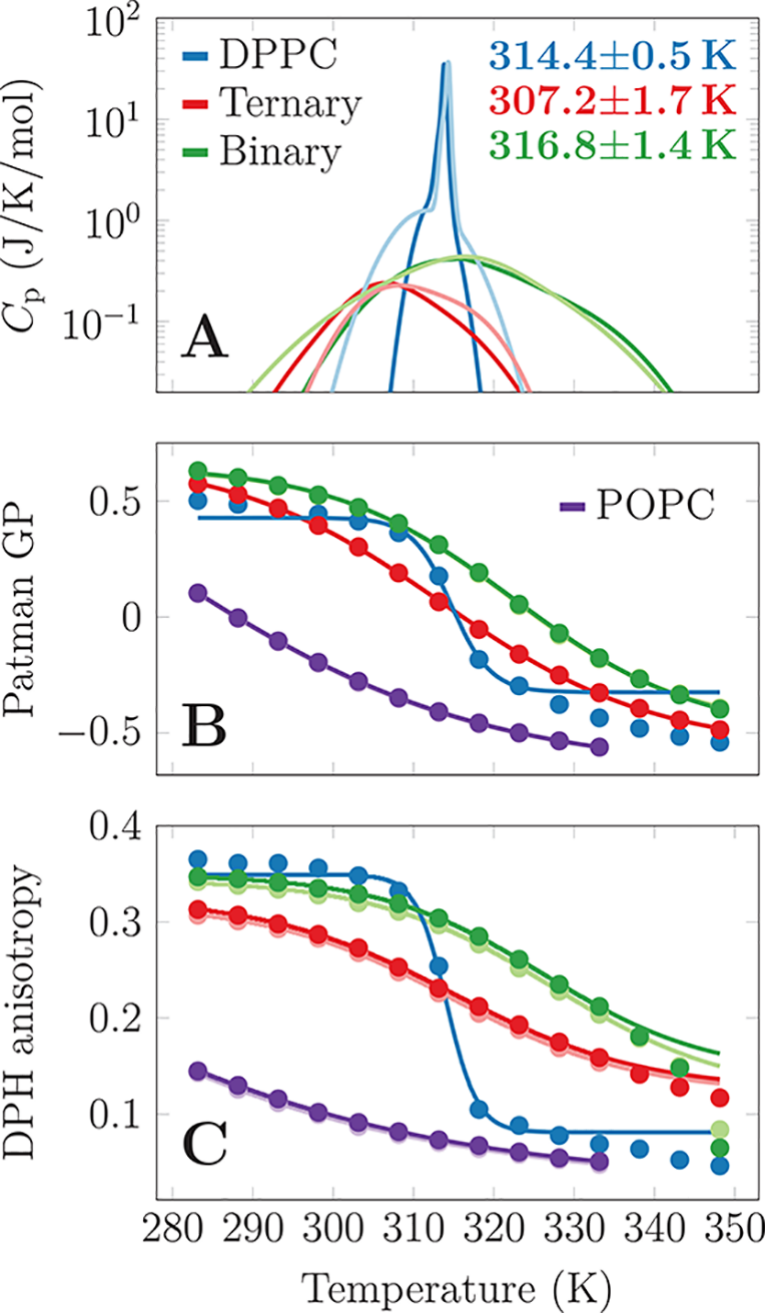
Results from differential scanning calorimetry and fluorescence spectroscopy. (A) Specific heat capacity. The profiles were fitted by two or three Gaussians, and the fitted data are shown here with a logarithmic *y* axis for easier comparison. The darker and lighter curves correspond to cooling and heating scans, respectively. The original data as well as the fits are shown in Figure S10, and the parameters obtained from the analysis of the DSC curves are available in Table S2. (B) Generalized polarization parameter with Patman. Two independent measurements were performed for each sample (except for DPPC), yet they overlap. The data for *binary* and *ternary* mixtures show a change of curvature (inflection point); yet POPC lacks it, as it is in the L_d_ phase at all measured temperatures. The spectra are shown in Figure S11, and the parameters extracted from fitting a modified Boltzmann curve are available in Table S3. (C) DPH anisotropy. Two samples were again measured for compositions other than DPPC, and they are almost identical. The spectra are shown in Figure S12 and the fitting parameters from a modified Boltzmann curve in Table S4. DPH anisotropy becomes wavelength-dependent at higher temperatures (see Figure S12), leading to the deviation of the measured data from the fit. Therefore, points measured above 333 K were excluded from the fitting process.

The second fluorescence technique we used is based on a different principle and provides complementary information about the lipid hydrocarbon chain region. Diphenylhexatriene (DPH) is a rod-like hydrophobic fluorescent probe with simple geometry and directional emission of polarized light. It orients along the lipid hydrocarbon chains and measures their order and dynamics—both indicative of the membrane phase.^47^ Our previous studies using time-resolved anisotropy measurements demonstrated great sensitivity of DPH to the ordering effect of different amounts and different species of sterols in the lipid bilayer.^48,49^

The temperature dependence of the anisotropy emission spectra of DPH is shown in Figure S12. Similarly to GP discussed above, the average anisotropy values (Figure 4C) show a gradual decrease in anisotropy upon a temperature increase for both the *ternary* and *binary* mixtures. This change reflects a decreased order and increased dynamics of the lipid acyl chains. Noteworthy, the qualitative similarity of the results obtained using Patman GP and DPH anisotropy reassures us that the observed changes can be detected both in the bilayer core, but also closer to the headgroup region, and are not limited to a single fluorescent probe or detection method used. The data can again be fitted by the modified Boltzmann sigmoidal functions (Figure 4B). Interestingly, Patman reports the changes to take place at a slightly lower temperature—particularly for the *binary* system—indicating that the melting of the hexagonal packing of DPPC chains initiates from the carbonyl region probed by Patman, before it proceeds to the chain region probed by DPH. This finding is in agreement with the previously observed initial melting of the glycerol backbone.^16,17^ All of the fitting parameters are provided in Table S4. Please note that for higher temperatures (>340 K), DPH does not provide trustworthy data for DPPC and the *binary* mixture. This is likely caused by the probe relocation and can be detected by the wavelength-dependent anisotropy data (Figure S12C,E; see also ref (50)). This is not observed in POPC nor in the *ternary* mixture.

Summarizing, we have provided a molecular-level view into the structures of the L_o_ phase across a range of temperatures in a *binary* DPPC/CHOL mixture and a *ternary* DPPC/DOPC/CHOL mixtures. Both mixtures are expected to be in the L_o_ phase across the studied temperature range from 293 to 333 K. Still, we discovered two different regimes separated by a crossover at either *T*_co_^ter^ ≈ 308 K or *T*_co_^bin^ ≈ 318 K; yet, no lipid demixing was observed.

The hexagonally packed clusters of DPPC chains that are present at low temperatures melt upon heating at *T*_co_, which affects many structural and dynamic membrane properties. Interestingly, the temperature dependencies of these properties were different not only below and above the crossover temperature but also between the *binary* and *ternary* mixtures. Still, the properties of the two mixtures converged toward each other at low and high temperatures. The properties of the *binary* mixture were more different on the two sides of the crossover temperature, highlighting that the presence of DOPC is able to buffer many properties in the *ternary* mixture. Still, all studied properties change continuously with temperature, and no radical changes akin to a first-order phase transition were observed.

On the experimental side, both the DSC scans and the fluorescence measurements suggest that a structural transition takes place at temperatures close to *T*_co_ obtained from simulations. Moreover, both experimental approaches indicate that this transition is more subtle than a proper first-order phase transition. Despite the use of probes in fluorescence experiments, the limitations of the simulation models, and the possible differences in the vesicles generated in two laboratories, the estimated transition temperatures differ by a mere few K. Moreover, all approaches systematically suggest that the *T*_co_ values differ by ∼10 K between the *ternary* and *binary* mixtures. Despite this difference, the behavior of the *ternary* and *binary* mixtures is strikingly similar.

Curiously, the two very different molecular structures at different temperatures—one with gel-like hexagonally packed DPPC clusters among more fluid regions and the other consisting of a fully fluid structure with only slightly preferred interactions among the components—are both fluid: The characteristic time scales of lipid rotational and translational motions differ by only two orders of magnitude and thus all fall within the L_o_-like regime between the L_d_ and gel phases. The L_o_-like high values for the deuterium order parameters are also reproduced by our simulations at all the studied temperatures, i.e., by structures where CHOL either is in direct contact with the chains or excluded from the hexagonally packed clusters of DPPC chains. Therefore, it seems that the two distinct molecular structures where CHOL has two very different roles—either ordering nearby-residing DPPC chains (above *T*_co_) or lubricating the space between the gel-like DPPC clusters (below *T*_co_)—lead to similar average behaviors that are indistinguishable by many experiments.

CHOL induces the L_o_/L_d_ coexistence in mixtures with a low-*T*_m_ lipid and a high-*T*_m_ lipid by melting the gel phase (formed mainly by the high-*T*_m_ lipid) that would exist in the mixture in the absence of CHOL. However, this “melting” only corresponds to breaking the hexagonally packed lipids into smaller and mobile clusters. While this leads to L_o_-like behavior, a large fraction of the lipid chains are still in the gel-like highly ordered yet untilted state. Only above *T*_co_ do the hexagonally packed clusters really melt, leading to proper lipid mixing. Curiously, coexisting L_o_ and L_d_ phases in phase-separated DPPC/DOPC/CHOL liposomes mix above 308 K (= *T*_co_^ter^).^27^ Thus, it seems likely that the hexagonally packed clusters are required to sustain the L_o_/L_d_ coexistence. Indeed, CHOL cannot induce this coexistence from any uniform L_d_-phase mixture above *T*_m_ of the high-*T*_m_ lipid, supporting this idea.

The differences between the two molecular-level structures of the L_o_ phase in these two temperature regimes might play key roles in biomembranes. Importantly, on the basis of our findings, the use of L_o_/L_d_ coexistence observed in synthetic or giant plasma membrane vesicles at relatively low temperatures as a model for lipid rafts existing at body temperature should be questioned, as the structures of the ordered domains in these systems might be very different. This might explain the discrepancies in protein partitioning between *in vitro* studies exploiting phase-separated vesicles and indirect *in vivo* data. Synthetic and giant plasma membrane vesicles display L_o_/L_d_ coexistence only at room temperature or lower. In this temperature range, the L_o_ phase possibly contains clusters of very tightly packed lipid chains—a structure which is likely unable to solvate large transmembrane protein segments. Indeed, raft-associated proteins were found in the L_d_ phase in synthetic lipid mixtures containing sphingomyelin^29^ for which similar tight lipid chain packing has been observed.^51^ The effect of temperature is manifested by an increased tendency of peptides to partition to the ordered detergent-resistant membranes at 310 K as compared to 277 K.^38^ While this finding was originally associated with hydrophobic mismatch,^38^ it could as well result from changes in the molecular structure of these membranes.

Coexisting phases in plasma membrane-derived vesicles are compositionally and thus also structurally more similar than in their synthetic counterparts,^28,34,35,37^ suggesting that their L_o_ phases might not be as rigid as those in synthetic mixtures. Since the plasma membrane is compositionally complex,^52^ it is possible that critical fluctuations^39^ can assemble domains with varying lifetimes and compositions.^37^ In this picture, domains containing very long chain sphingolipids^52^ could still have a *T*_co_ that is higher than the physiological temperature, suggesting that both of the L_o_ structures described in this study could coexist in the plasma membrane.

## Supporting Information Available

Detailed methodology of the simulations and experiments. Additional results from simulations and experiments. (PDF)
Transparent Peer Review report available (PDF)

## Notes

Simulation inputs and outputs are available at the Zenodo repository at DOI: 10.5281/zenodo.4110687 and DOI: 10.5281/zenodo.4309026.
