# Supplementary material for: The Two Faces of the Liquid Ordered Phase
Source: J Phys Chem Lett. 2022 Feb 1;13(5):1307–13. doi: 10.1021/acs.jpclett.1c03712 (PMC8842317; doi:10.1021/acs.jpclett.1c03712)
Supplement: Supplementary file 1 — jz1c03712_si_001.pdf [file jz1c03712_si_001.pdf]

Supplementary Information for: The Two Faces of the Liquid Ordered Phase

Itay Schachter,[†,‡,¶] Riku O. Paananen,[§,‖,¶] Balázs Fábián,[†] Piotr Jurkiewicz,[*,⊥] and Matti Javanainen[*,†,#]

†*Institute of Organic Chemistry and Biochemistry of the Czech Academy of Sciences, Flemingovo nám. 542/2, CZ-16000 Prague 6, Czech Republic*

‡*Institute of Chemistry, the Fritz Haber Research Center, and the Harvey M. Kruger center for Nanoscience & Nanotechnology, The Hebrew University, Jerusalem 9190401, Israel*

¶*These authors contributed equally.*

§*Department of Chemistry, University of Helsinki, FI-00014 University of Helsinki, Finland*

‖*Department of Ophthalmology, University of Helsinki and Helsinki University Hospital, FI-00014 University of Helsinki, Finland*

⊥*J. Heyrovský Institute of Physical Chemistry of the Czech Academy of Sciences, Dolejškova 2155/3, CZ-18223 Prague 8, Czech Republic*

#*Institute of Biotechnology, University of Helsinki, FI-00014 University of Helsinki, Finland*

E-mail: piotr.jurkiewicz@jh-inst.cas.cz; matti.javanainen@helsinki.fi

Contents

S1 List of Studied Systems **S2**

S2 Details on Simulation Methods **S2**

S3 Details on Simulation Analyses **S4**

S4 Details on Differential Scanning Calorimetry **S8**

S5 Details on Fluorescence Spectroscopy **S8**

S5.1 Patman Generalized Polarization S8

S5.2 DPH Anisotropy S9

S5.3 Materials S9

S5.4 Liposome Preparation S10

S5.5 Spectroscopic Methods S10

S6 Fluorescence Data Analysis **S11**

S7 Additional Results **S13**

S7.1 Simulations S13

S7.2 Differential Scanning Calorimetry S21

S7.3 Fluorescence Spectroscopy S23

References **S26**

Table S1: Compositions of the studied systems. DOPC stands for 1,2-dioleoyl-sn-glycero-3-phosphocholine, DPPC for 1,2-dipalmitoyl-sn-glycero-3-phosphocholine, and POPC for 1-palmitoyl-2-oleoyl-sn-glycero-3-phosphocholine. In the "Method" column, we indicate whether simulations ("S"), fluorescence experiments ("F"), or calorimetry experiments ("C") were performed on the mixture. The rightmost column indicates the number of simulations performed, and the total simulation time. The 9+2 notation indicates that 9 simulations were performed at different temperatures, whereas additional simulations were performed for the ternary mixture at the lowest/highest temperature and with the initial structure taken from the final structure of the simulation at the highest/lowest temperature to ensure that hysteresis did not affect the obtained results in the simulation time scale.

System	DPPC	DOPC	Chol.	POPC	Method	Simulations
Ternary	55%	15%	30%	0%	S+F+C	(9+2)×1 µs
Binary	70%	0	30%	0%	S+F+C	9×1 µs
DPPC	100%	0%	0%	0%	S+F+C	–
POPC	0%	0%	0%	100%	F	–
Ternary-15	67%	18%	15%	0%	C	–
Binary-15	85%	0%	15%	0%	C	–
DOPC-DPPC	79%	21%	0%	0%	C	–

S1 List of Studied Systems

S2 Details on Simulation Methods

The compositions of the simulated systems are provided in Table S1. All simulated systems had a total of 512 lipids, and were solvated by 50 waters per lipid (total of 25600 molecules) and 61 Na^+ and Cl^- ions.

The CHARMM36 model for phospholipids[1] and cholesterol[2] was used together with the CHARMM-specific TIP3P water model.[3] The recommended simulation parameters[4] provided by CHARMM-GUI for the GROMACS simulation package[5,6] are as follows: The leap-frog integrator was used with a time step of 2 fs. Buffered Verlet lists were used to keep track of close neighbours.[7] The Lennard-Jones potential was cut-off at 1.2 nm, and the forces switched to 0 between 1.0 and 1.2 nm. The smooth particle mesh Ewald (PME) algorithm was used to calculate the electrostatic interactions.[8,9] The membrane and the solvent were

separately coupled to a Nosé–Hoover thermostat[10,11] with a time constant of 1 ps. The pressure was coupled semi-isotropically to the Parrinello–Rahman barostat[12] with a time constant of 5 ps, a compressibility of 4.5×10^{-5} bar^{-1}, and a reference pressure of 1 bar. The LINCS algorithm[13] was used to constrain bonds involving hydrogens.

The simulations of the membranes with *binary* and *ternary* lipid mixtures were performed at 293, 298, 303, 308, 313, 318, 323, 328, and 333 K. All these simulations were 1 µs long, the trajectories were saved every 100 ps, and the first 100 ns was omitted in the analyses. To ensure that the systems had relaxed to their equilibrium conformations and were not affected by the initial conditions, we repeated the simulations of the *ternary* mixture at 293 K and 333 K, initiating the simulations from the final structures of the simulations performed at 333 K and 293 K, respectively. The input and output files are freely available online, and the DOIs pointing to these data are provided in the main text.

S3 Details on Simulation Analyses

The simulation analysis was done by tools bundled in Gromacs and in-home tools written in Matlab r2016b (The MathWorks, Inc., Natick, Massachusetts, United States) and the Python programming language (Python Software Foundation, https://www.python.org/). Details on the analyses are provided in the SI and implementations are available from authors upon request.

Area per Phospholipid We calculated the area per phospholipid (APPL) simply by dividing the total area of the simulated membrane by the number of phospholipids in a single leaflet (178). The box dimensions were extracted by `gmx energy` bundled with the GROMACS simulation package.

Lipid Chain Tilt The distribution of the tilt angle of the *sn*-1 chain of DPPC was extracted by `gmx gangle` bundled with the GROMACS simulation package. The angle was

defined between the vector connecting the C31 and C316 atoms (1st and last carbon atoms in the *sn*-1 chain) and the direction normal to the membrane, *i.e.* the z axis in the simulation box. The obtained distributions followed the Γ distribution, and their mean value was extracted.

Lateral Diffusion Coefficients The lateral diffusion coefficients were extracted for the lipid centers of mass (COMs). The COM trajectory was first generated by `gmx traj` bundled with the GROMACS simulation package. The mean squared displacement (MSD) data were extracted from both leaflets, and fitted by MSD $= 4D\Delta$, where D is the lateral diffusion coefficient and Δ is the lag time, indicating averaging over time. Additionally, the MSD data were averaged over molecules within a leaflet. For the MSD calculation, the lateral leaflet drift was eliminated. The mean value and the difference between the diffusion coefficient values extracted from the two leaflets were reported as the value and the error estimate, respectively. No correction due to periodic boundary conditions[14] was performed, as the viscosities of the membranes were not known.

Rotational Dynamics The rotational dynamics of a lipid molecule was described by the characteristic decay time of the orientational autocorrelation function of the vector connecting the C2 and C3 carbons of the glycerol backbone, *i.e.* the carbons to which the two acyl chains are esterified. The time-averaged normalized autocorrelation function was fit from the beginning until the time at which it had decayed to 0.1 with a double exponential. The larger of the two time constants is reported, as it corresponds to proper molecular rotation, whereas the smaller of the two describes only the rattling on the short time scale.

Hexatic Order Parameter The hexatic order parameter was calculated using Freud library[15] for the in-plane coordinates of the C10 atoms of cholesterol, and C210 C310 atoms of DPPC and DOPC lipids (10th carbons in the two acyl chains). The norm of the complex-valued order parameter obtained from Freud was defined as the hexatic order parameter of

individual lipids in a given frame. The analysis was done separately on each leaflet. The average values were calculated for each moiety. Standard errors were evaluated using the block averaging method.

Neighbor Persistence The neighbor persistence after a lag time t ($:= p(t)$) as the average number of the 6 nearest-neighbor lipids that were not exchanged during the lag time t. $p(t)$ was calculated for DPPC lipids whose 6 nearest-neighbors were other DPPC lipids at an initial frame as representing lipids within the hexagonally-packed DPPC clusters. The standard errors (STEs) were evaluated with the block averaging method. The persistence times were obtained from a linear fit to the decay curves in the log–linear scale (exponential in linear scale). The fits were performed using the SciPy library.[16] For error estimation, upper and lower bounds for the decay rates were taken as the fitted decay rates for $p_{\text{lower}}(t) = p(t) + STE[p(t)]$ and $p_{\text{upper}}(t) = p(t) - STE[p(t)]$.

Local Environments The local enviroments of lipids outside the DPPC clusters were evaluated. First, DPPC clusters were defined using DBSCAN algorithm[17] (ϵ =0.7 nm and a minimum of 6 neighbors) on each leaflet separately, with in-plane positions of C310 C210 atoms of DPPC (10th carbon atoms of each DPPC chain). A clustering value v was given to each tail ($v = 1$ if the tail found to be within a cluster, otherwise 0). The v value was averaged over the 2 tails of each lipid and then temporally smooth by uniform filtering with width of 50 ns. Finally, lipids with instantaneous $v > 0.2$ are identified as in a DPPC cluster. For each frame, the instantaneous local environment of each lipid outside of a DPPC cluster was calculated by the fraction of each component—omitting lipids in a DPPC cluster—at a radius of 1 nm from it. The local environment is than averaged both temporally and by lipid type. The local environment is then compared to the expected environment in the case of ideal mixing, which is the average composition (up to a negligible bias due to system's finite size relative to the presented deviation from ideal mixing) of the leaflet after removing the DPPC clusters.

Deuterium Order Parameter The deuterium order parameters (see Ref. 18 for definition) were extracted for the $sn-2$ chain of DPPC using the `gmx order` tool bundled with the GROMACS simulation package. This tool uses the carbon positions to extract the order parameter, hence it provides values for carbons 2–15 along the 16-carbon chain.

Classification to Core, Edge, and Free Points by Clustering Similarly to the clustering method for the Local Enviroments (see the respective paragraph above), the assignment of lipid to being in a cluster was obtained, the only difference was that v was not averaged over the 2 tails of each DPPC lipid, thus making the assignment per-tail instead of per-lipid. After this assignment, DPPC tails that were not part of a cluster are classified as "Free". Then, the remaining DPPC tails are classified separately as "Core" or "Edge", depending on if the DBSCAN classified them as core points or as edge points more often over a smoothing window of 50 ns.

Analysis of Classified DPPC Chains After classification, the average deuterium order parameter (S_{CD}) and chain tilt angle were calculated for each classification group (core, edge, and free). S_{CD} was calculated from definition based on the C–H bond for each carbon in the chain with bonded to exactly 2 hydrogen bonds, and then averaged throughout each chain (see Ref. 18 for definition). The chain tilt angle was defined as the angle between the bilayer normal ($\pm\hat{z}$) and the vector connecting the 1_{st} and 16th carbons of both acyl chains (C21 & C216 and C31 & C316 atom pairs).

Density Profiles The density profiles of the phosphorus atoms of DPPC and DOPC, as well as the hydroxyl oxygen atom of cholesterol were calculated using `gmx density` bundled with the GROMACS package. The simulation box was divided into 200 bins along the direction normal to the membrane, *i.e.* the z axis. The profiles were centered based on the lipid center of mass, and symmetrized. The locations of the density peaks were obtained by fitting the data corresponding to one leaflet with a Gaussian function.

S4 Details on Differential Scanning Calorimetry

Liposome samples were prepared using DPPC and cholesterol obtained from Sigma–Aldrich (Steinheim, Germany) and DOPC obtained from Avanti Polar Lipids (Alabaster, AL, USA). Initially, lipid mixtures were prepared in chloroform solution and transferred into glass test tubes. Chloroform was evaporated under nitrogen flow and the samples were then held in a vacuum dessicator for 3 h to remove the residual solvent. Ultrapure water (Maxima MK3, USF Elga, Bucks, UK) was added to the dried lipid film and the lipid was hydrated above the transition temperature of the lipid mixture at 323 K to 343 K accompanied by vigorous agitation to produce 0.8 mM solutions of multilamellar vesicles. To produce large unilamellar vesicles, the multilamellar vesicle solutions were then extruded 21 times through a Isopore 100 nm PC membrane filter using Avestin LiposoFast-Pneumatic extruder (Ottawa, Canada) connected to a water bath to to maintain the temperature of the samples above the transition temperature during extrusion. The unilamellar vesicle mixtures were stored at 277 K until measured. Differential scanning calorimetry measurements were performed using Microcal PEAQ-DSC (Malvern Panalytical, Malvern, UK). The samples were degassed prior to measurements and heating and cooling scans were performed at a rate of 60 K/h using high gain. Background scans were performed with ultrapure water and subtracted from each scan.

S5 Details on Fluorescence Spectroscopy

S5.1 Patman Generalized Polarization

Patman is a polarity-sensitive fluorescent probe stably located in numerous lipid membranes. The charge transfer over the naphthalene ring provides large change in fluorophore dipole moment upon excitation, resulting in ≈140 nm Stokes shift in its fluorescence.[19] The Stokes shift depends on the environment polarity, but is also very sensitive to the mobility of the polar

moieties in the vicinity of the probe.[20] Anchored by hydrophobic tail to the membrane interior, but also by the quaternary ammonium group to the lipid headgroups, the fluorophore of Patman is exceptionally stably located in neutral lipid bilayers[21] (Fig. S11A). The emission spectra of this probe are, thus, sensitive to membrane hydration and, even more, the mobility of the hydrated lipid carbonyls. Since both of these parameters are strongly affected by the phase state of the lipids, the temperature scans using Patman and similar probes (*e.g.*, Prodan, Laurdan) allow to easily determine the phase transitions in lipids. In fact, the characteristic emission wavelengths of Patman in lipid gel and liquid disordered phases (420 nm and 495 nm, respectively) were used to define the so-called generalized polarization parameter (see below).

S5.2 DPH Anisotropy

DPH (diphenylhexatriene) is a rod-like hydrophobic fluorescent probe that locates in the core of lipid membranes (Fig. S12A).[22,23] It is predominantly oriented perpendicular to the membrane surface and along the lipid hydrocarbon tails. Its simple geometry and directional emission of polarized light is often utilized to report on the order and the dynamics of the lipid tails. Since lipid thermotropic transitions are often dominated by the chain melting, the use of DPH is a good choice for characterizing the phase state of a lipid bilayer. Herein, we measured steady-state anisotropy emission spectra of DPH embedded in the lipid bilayer of unilamellar vesicles to probe the phase behavior of lipids using the same lipid mixtures and temperatures as in the case of the experiments in which Patman was used.

S5.3 Materials

Phosphatidylcholines: DOPC, DPPC, and POPC were ordered from Avanti Polar Lipids, Inc. (Alabaster, USA). Fluorescent probes: DPH (1,6-diphenyl-1,3,5-hexatriene), and Patman (6-hexadecanoyl-2-(((2-(trimethylammonium)ethyl)methyl)amino)naphthalene chloride) were purchased from Invitrogen (Eugene, OR, USA). Hepes (4-(2- hydroxyethyl)-1-piperazineethanesulfonic

acid) buffer, KCl, and organic solvents of spectroscopic grade were supplied by Merck (Darmstadt, Germany). All chemicals were used without further purification.

S5.4 Liposome Preparation

Extruded liposomes were prepared as follows; Appropriate volumes of chloroform solutions of lipids and methanol solution of one of the fluorescent probes were mixed transferred to glass vials using glass syringes (Hamilton Company Inc., NV, USA). Organic solvents were evaporated under gentle stream of nitrogen, while heated to room temperature with a water bath, and then for 1 hour under vacuum. Dry lipid film was hydrated with buffer composed of 10 mM Hepes, pH=7.4 (KOH), 150 mM KCl, and Mili Q water (Milipore, USA). Formation of multilamellar vesicles was accelerated with heating the sample and vortexing using small glass beads. Extrusion of all the samples through polycarbonate filters with a nominal pore diameter of 0.1 µm was performed >333 K with hand extruder LipoFast-Basic (Avestin, Ottawa, Canada). We used 25 extrusion cycles. Measurements were completed within 24 h after extrusion. The size distribution of the vesicles was measured by means of dynamic light scattering after 24±2 h using Zetasizer Nano ZS (Malvern Instruments Ltd., Worcestershire, UK). The mean vesicle diameter varied slightly within 125–140 nm. Final lipid concentration was 0.2 mM, and the content of fluorescent probes was 0.5 mol%.

S5.5 Spectroscopic Methods

Dispersions of the extruded vesicles were transferred to 1.5 mL quartz spectroscopic cuvettes and equilibrated before each measurement at desired temperature for 15 min. Temperature was kept constant within 0.5 K. Temperature scans were performed from 283 to 348 K with 5 K steps. All fluorescence measurements were performed using FS5 spectrofluorometer (Edinburgh Instruments Ltd., Livingstone, UK).

Patman-containing samples were excited at 360 nm and the emission spectra were collected from 380 to 600 nm. Excitation polarizer was set to vertical orientation and emission

one to magic angle. DPH-containing samples were excited at 358 nm and the anisotropy spectra were collected from 400 to 500 nm using build-in polarizers. Both for Patman and DPH samples, 5 nm excitation and 3 nm emission monochromator slits were used, and three consecutive spectra were summed up. All samples were checked for changes in optical density using absorbance detection built in the FS5 spectrofluorometer. No significant changes in optical density were observed upon heating the samples from 283 to 333 K.

S6 Fluorescence Data Analysis

Excitation generalized polarization was calculated from the emission spectra recorded for Patman using the following formula:

$$\mathrm{GP} = \frac{F_{420} - F_{495}}{F_{420} + F_{495}}, \tag{1}$$

where F_{420} and F_{495} are the mean fluorescence intensities measured 417 to 423 nm, and from 492 to 498 nm, respectively.[24]

Steady-state DPH anisotropy spectrum was calculated as:

$$\mathrm{r}(\lambda) = \frac{F_{\mathrm{VV}}(\lambda) - F_{\mathrm{VH}}(\lambda)}{F_{\mathrm{VV}}(\lambda) + F_{\mathrm{VH}}(\lambda)} \cdot \frac{F_{\mathrm{HH}}(\lambda)}{F_{\mathrm{HV}}(\lambda)}, \tag{2}$$

where λ in an emission wavelength, and the two-letter subscripts of fluorescence intensities F denote orientation of the excitation and emission polarizers (H for horizontal, V for vertical), respectively.[25] Mean anisotropy values were calculated from $r(\lambda)$ for λ from 430 to 470 nm.

Both GP and $\overline{r}$ values plotted as a function of temperature and fitted with modified Boltzmann sigmoidal function in the form of

$$y(T) = \frac{y_0 - y_\infty}{1 + \exp\left(\frac{T - T_0}{\alpha}\right)} + y_\infty, \tag{3}$$

where y_0 and y_∞ are the equilibrium values of the dependent variable y before and after the transition, T is temperature, T_0 is critical temperature, α is the slope of y at the transition (a measure of the rate of transition). The Boltzmann sigmoidal function was first normalized ($y_0 = 0$, $y_\infty = 1$) and then its first derivative was calculated as

$$\frac{\mathrm{d}y(T)}{\mathrm{d}t} = \frac{\exp\left(\frac{T-T_0}{\alpha}\right)}{\alpha\left[1+\exp\left(\frac{T-T_0}{\alpha}\right)\right]^2}. \tag{4}$$

Uncertainty of the formula (Eq. (4)) was calculated from the standard variance formula based on the partial derivatives of the function commonly used for the calculation of the propagation of uncertainties.[26] Standard errors obtained for the mean of only two measurement results were corrected using Student–Fisher coefficient 1.837. Confidence interval of 68% was chosen in all calculations.

S7 Additional Results

S7.1 Simulations

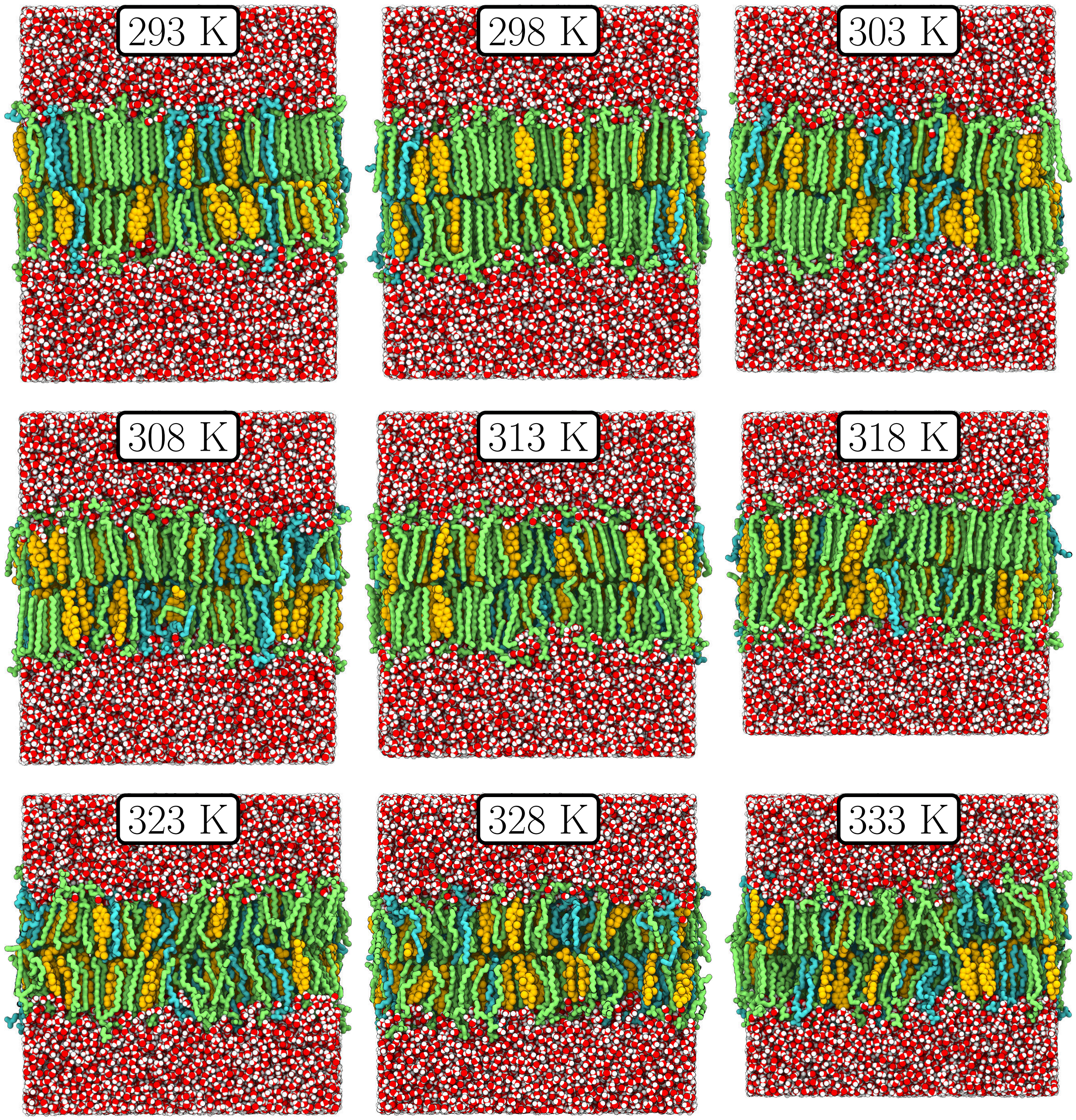

Figure S1: Final structures of the *ternary* mixtures at 293 K–333 K. Colors as in Fig. 1 in the main text. The chains seem to melt at around 308 K.

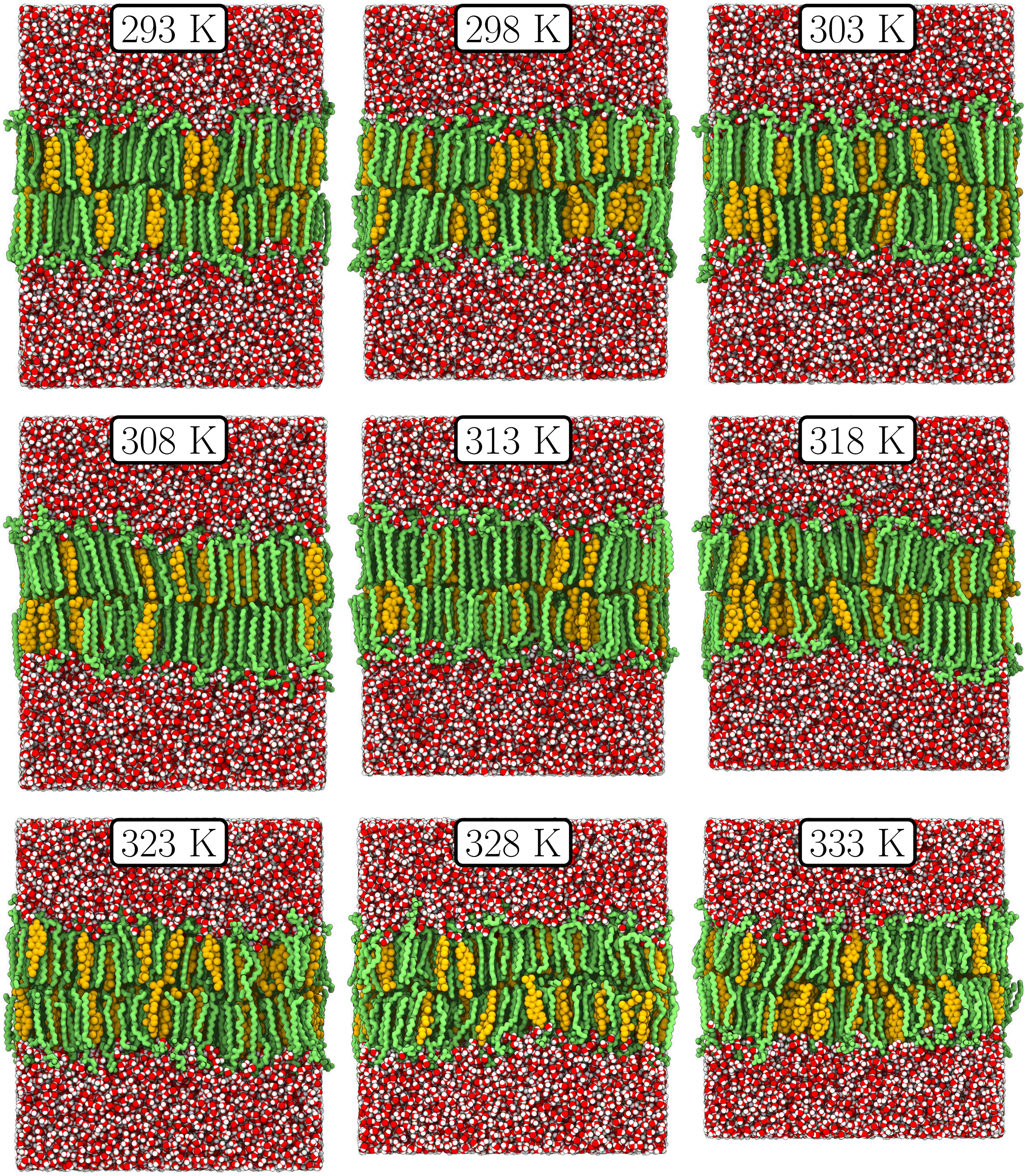

Figure S2: Final structures of the *binary* mixtures at 293 K–333 K. Colors as in Fig. 1 in the main text. The chains seem to melt at around 318 K.

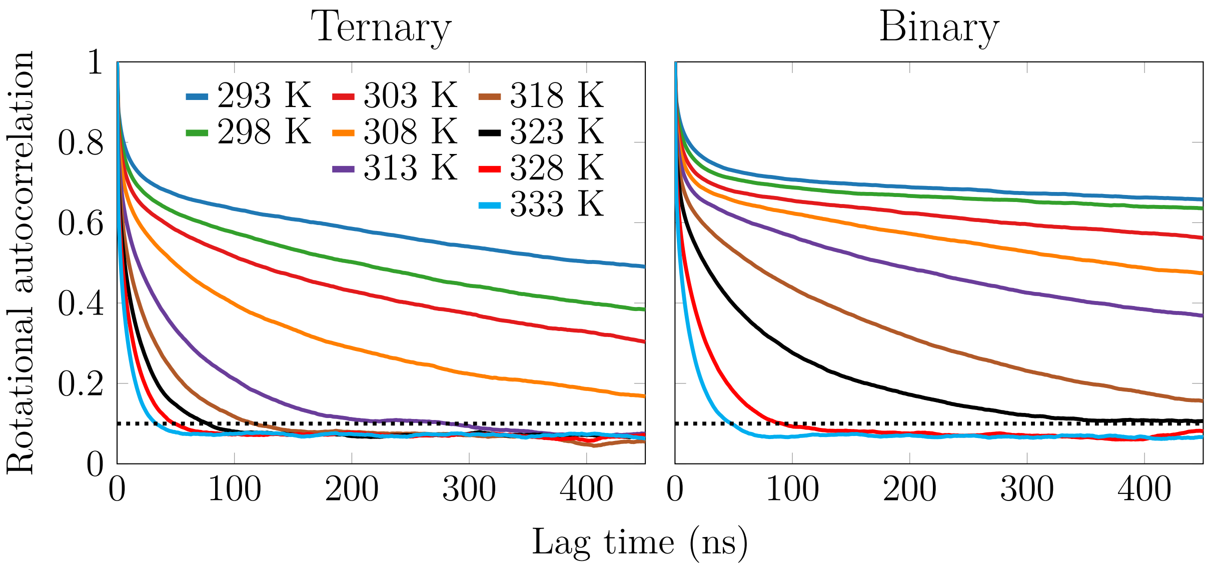

Figure S3: Rotational autocorrelation function of the vector connecting the C2 and C3 atoms in the glycerol backbone of DPPC. The dotted lines indicate the value of 0.1, up to which the double exponential fits were performed.

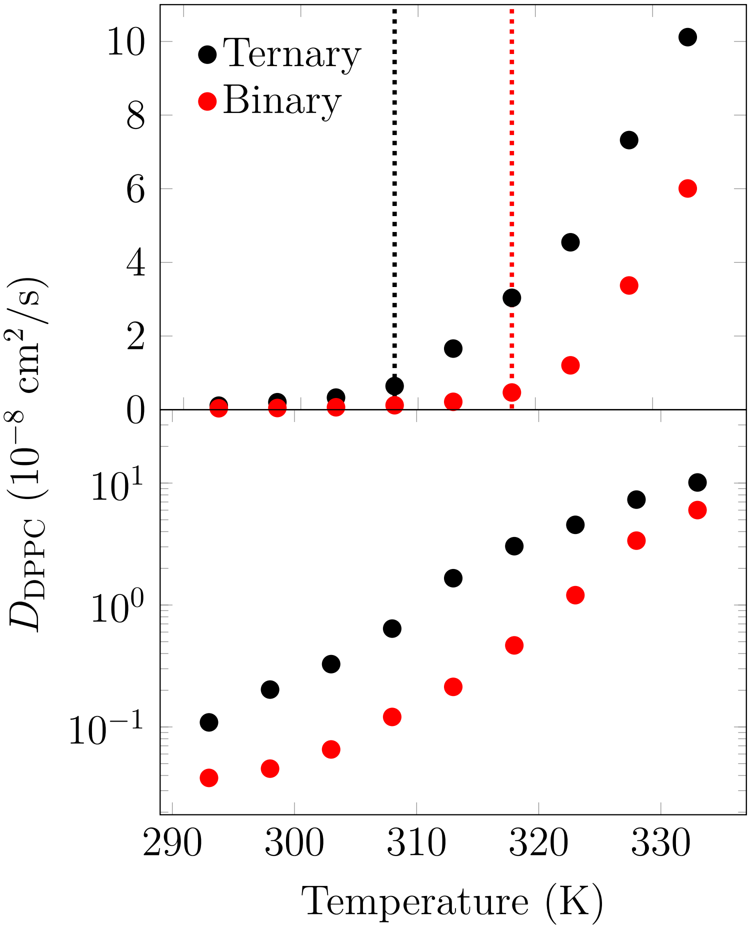

Figure S4: Diffusion coefficients of DPPC lipids in the *binary* and *ternary* mixtures in linear (top) and logarithmic scales (bottom). The dotted lines show the change in behavior at the crossover temperatures of ∼308 K (*ternary* mixture) or ∼318 K (*binary* mixture), as described in the main text. Gel phase values for DPPC are 10^{-11} cm$^2/s$, *i.e.* ≈2 orders of magnitude smaller than what we observe for DPPC in the mixtures at 293 K.[27]

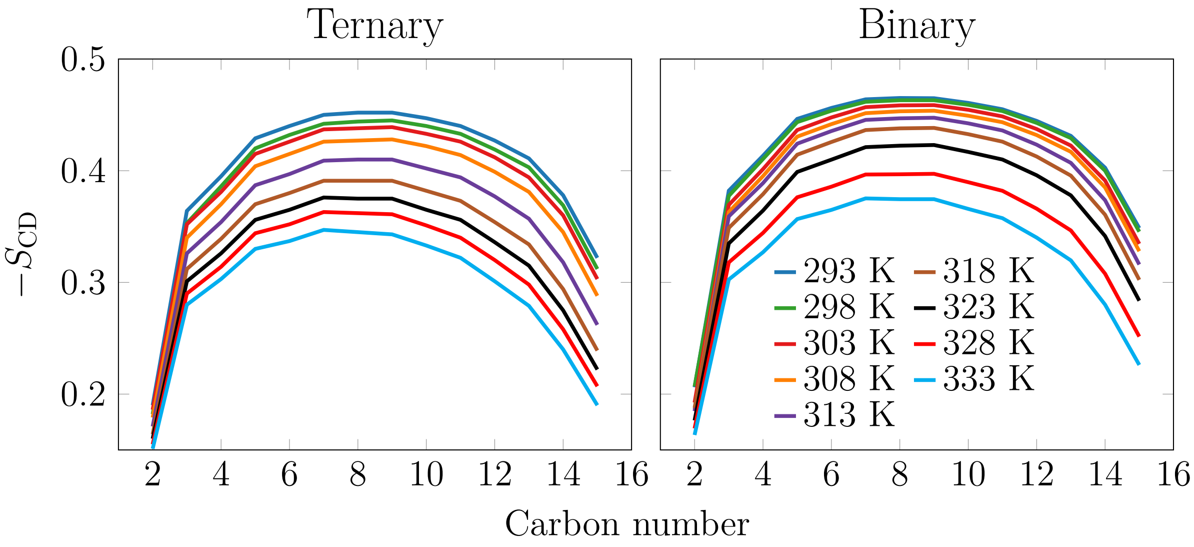

Figure S5: Deuterium order parameter profiles of the *sn*-2 chain of DPPC in the *ternary* (left) and *binary* (right) lipid mixtures.

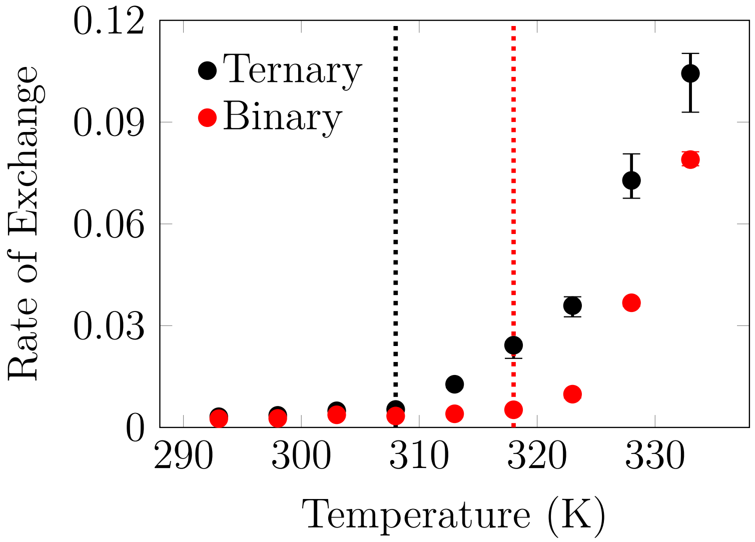

Figure S6: The rate of lipid neighbor exchange. In addition to the existence of hexagonally packed regions (see main text), we also evaluated the stability of the lipid neighbourhoods inside those regions by calculating the neighbour persistence rates for DPPC lipids with all initial 6 nearest neighbors being DPPC lipids as well—as expected by lipids inside the clusters—at which the initial nearest neighbors first get replaced by others. These values, plotted here, again show a rapid increase in this rate at temperatures above 308 K and 318 K for *ternary* and *binary* mixtures, indicating that the hexagonally-packed neighbourhoods are dissolved and lipids have more freedom to mix. Still, the lipids are not ideally mixed in the fluid regions (with hexagonally-packed DPPC-only regions omitted) either. Instead, we find that in these freely diffusing environments in the *ternary* mixture, the neighborhoods of DPPC are slightly enriched in cholesterol (by 22%) and somewhat (by 12%) in DOPC. In contrast, cholesterol neighbourhoods are depleted of DOPC (by –11%) as well as other cholesterol molecules (–14%). These tendencies are independent of temperature, indicating that these natural interaction tendencies[28] do not depend on the presence of hexagonally-packed DPPC clusters.

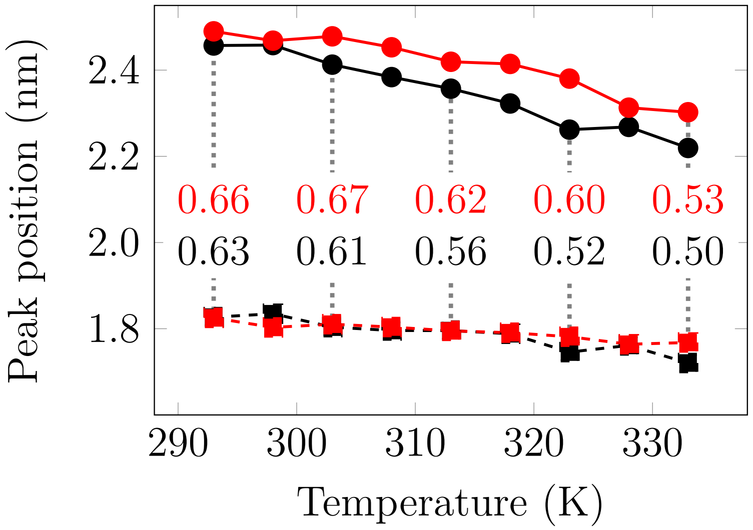

Figure S7: The positions of the maximum density of phospholipid phosphoruses (filled circles and solid lines) and CHOL oxygen atoms (filled squares and dashed lines) with respect to the membrane midplane. The numbers between the curves show the distance from CHOL oxygen to the lipid phosphorus atoms. The *binary* membrane thickness decreases linearly from 5.0 nm at 293 K to 4.6 nm at 333 K, T_{co}, in excellent agreement with scattering experiments on a similar membrane.[29] Despite membrane thinning, CHOL positioning remains fairly constant in both the *binary* and *ternary* mixtures, also in line with experiments.[30] The different temperature dependence of DPPC and CHOL positioning—each without any crossovers—causes the latter to move towards the headgroups at higher temperatures, as indicated by the labels.

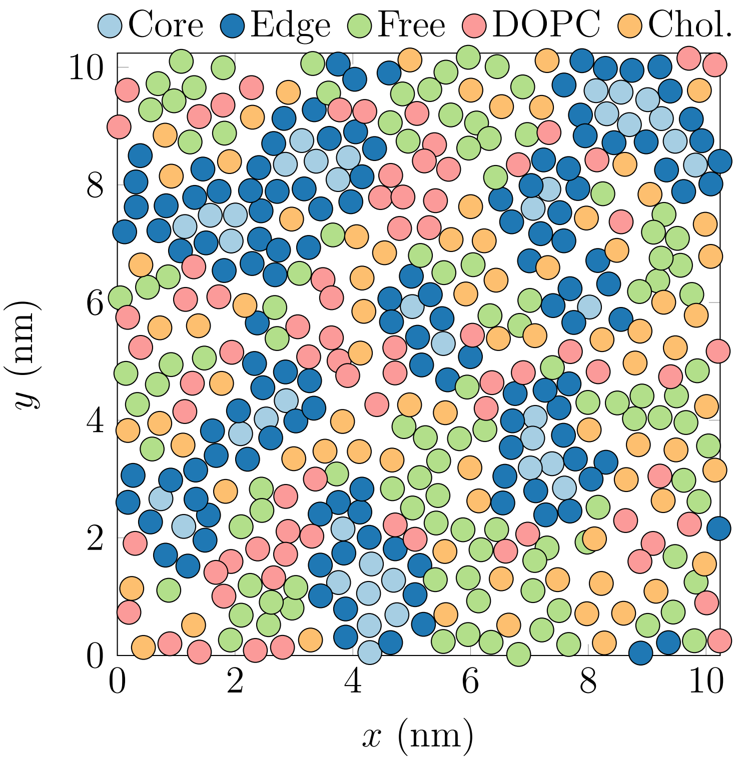

Figure S8: Example result of the DBSCAN clustering of the bilayer with a *ternary* lipid mixture simulated at 298 K. “Core” chains are surrounded by hexagonally-packed chains, “Edge” points are members of the hexagonally-packed clusters yet have both “Core” and “Free” chains as neighbors, and “Free” chains are not part of a hexagonally-packed cluster.

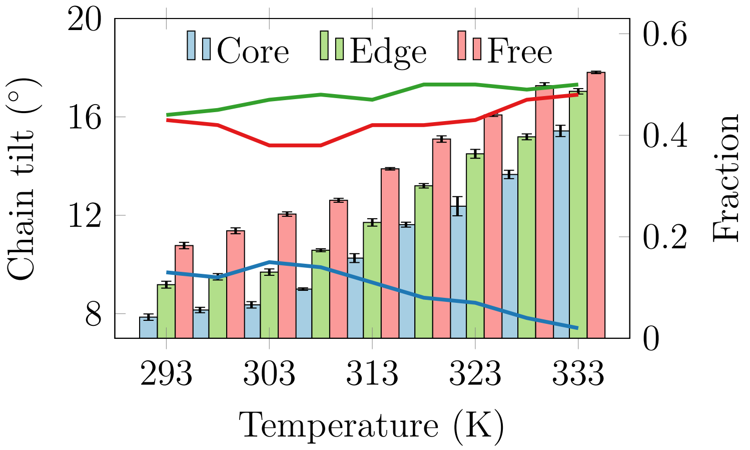

Figure S9: Tilt of the lipid chains (both *sn*-1 and *sn*-2) as a function of cluster identity are shown as bars. The lines show the fractions of each cluster identity.

S7.2 Differential Scanning Calorimetry

Table S2: Parameters of the DSC profiles. FWHM stands for "full width at half maximum".

System	Enthalpy (kJ/mol)		Peak position (K)		FWHM (K)	
	Heating	Cooling	Heating	Cooling	Heating	Cooling
DPPC	38.2	-36.1	314.7	314.1	0.6	0.6
Binary-15	18.6	-15.7	314.2	313.8	0.6	0.6
Binary	10.1	-9.2	317.5	316.1	19.9	19.4
DPPC–DOPC	26.6	-24.3	309.7	309.5	4.2	3.4
Ternary-15	9.7	-8.8	307.9	309.1	12.5	11.2
Ternary	3.7	-3.7	308.0	306.3	13.9	12.8

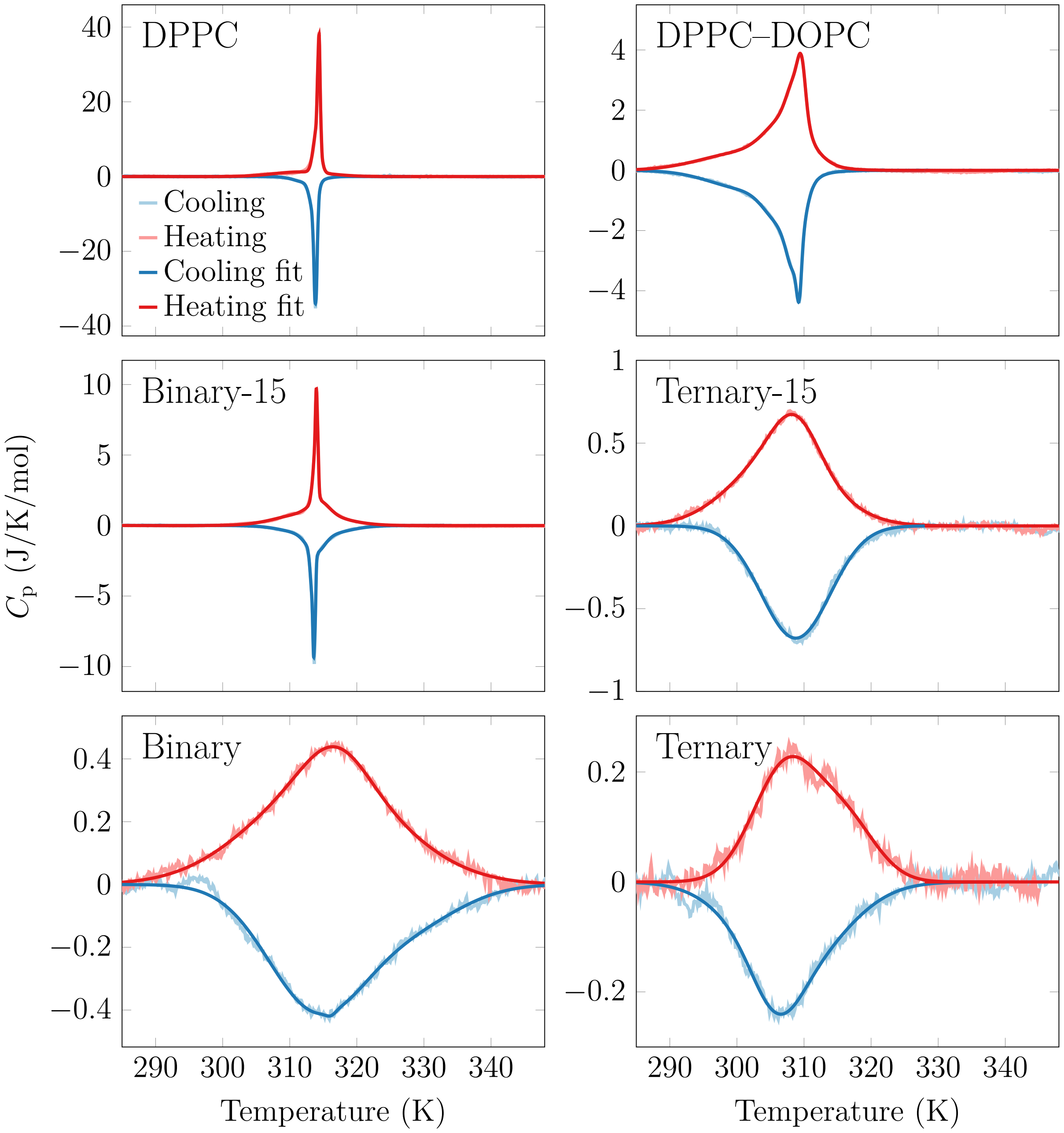

Figure S10: Experimental DSC curves and the fits of sums of Gaussians to them. The fitted data are shown in Fig. 5A in the main text.

S7.3 Fluorescence Spectroscopy

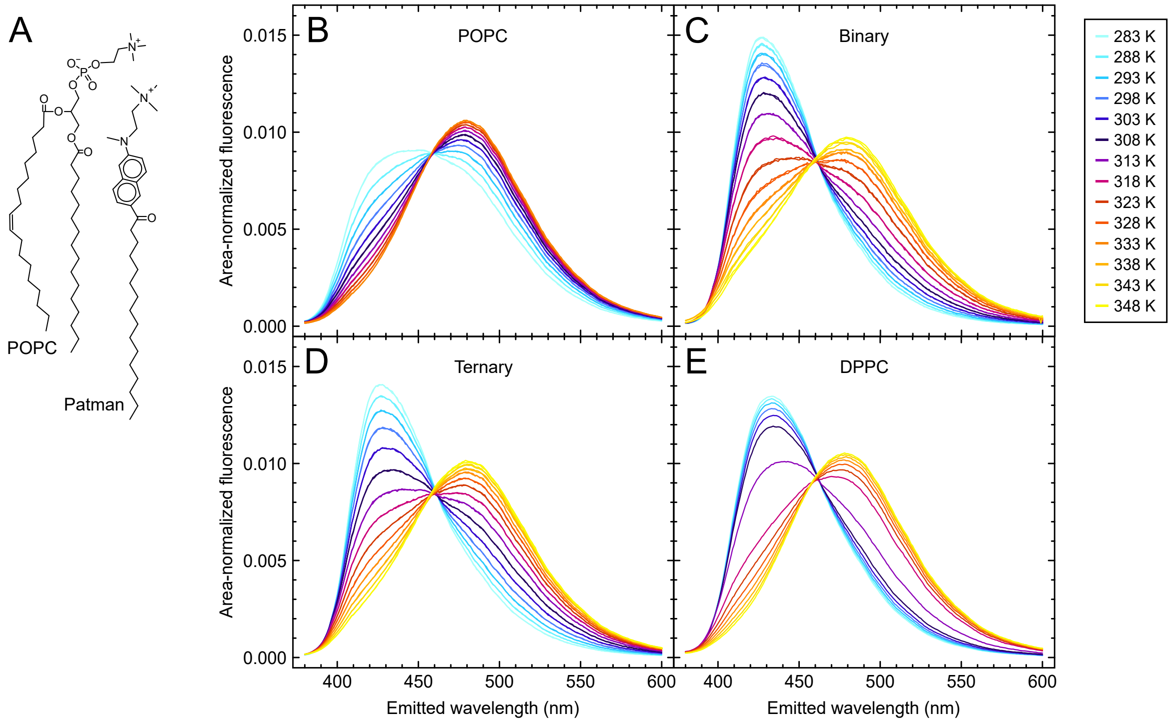

Figure S11: Fluorescence of Patman embedded in model lipid membranes. (A) Patman structure and its schematic location in POPC bilayer. Emission spectra of Patman in the membrane of large unilamellar vesicles composed of (B) POPC, (C) *binary* mixture, or (D) *ternary mixture* at different temperatures. The spectra measured for two independent samples are shown. Please note that the spectra for the two samples largely overlap.

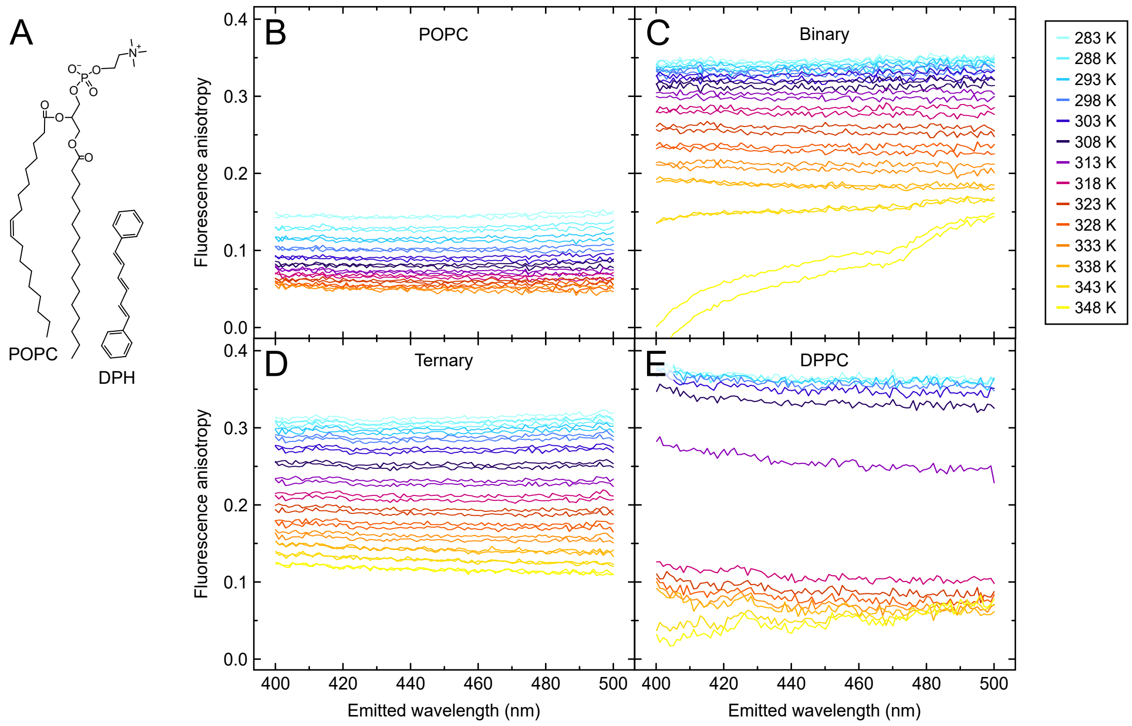

Figure S12: Fluorescence anisotropy of DPH embedded in model lipid membranes. (A) DPH structure and its schematic location in POPC bilayer. Steady state emission anisotropy spectra of DPH in the membrane of large unilamellar vesicles composed of (B) POPC, (C) *binary* mixture, or (D) *ternary* mixture at different temperatures. The spectra measured for two independent samples are shown.

Table S3: Parameters of the fits to Patman GP data (Eq. (4)).

System	T_0 (K)	α (K)	y_0	y_∞
Binary	322.0±0.4	11.0±0.4	0.65±0.01	−048±0.02
	322.6±0.4	11.4±0.4	0.66±0.01	−0.50±0.02
Ternary	313.5±0.3	13.0±0.3	0.69±0.02	−0.56±0.01
	313.5±0.3	13.2±0.4	0.69±0.02	−0.57±0.01
DPPC	314.7±0.4	2.5±0.4	0.43±0.02	−0.32±0.03

Table S4: Parameters of the fits to DPH anisotropy data (Eq. (4)).

System	T_0 (K)	α (K)	y_0	y_∞
Binary	327.4±1.7	11.1±0.8	0.34±0.01	0.12±0.02
	326.3±0.9	10.3±0.5	0.35±0.01	0.14±0.01
Ternary	314.8±0.6	11.4±0.7	0.32±0.01	0.12±0.01
	314.9±0.5	11.8±0.7	0.33±0.01	0.13±0.01
DPPC	314.1±0.3	1.9±0.3	0.35±0.01	0.08±0.01

References

(1) Klauda, J. B.; Venable, R. M.; Freites, J. A.; O'Connor, J. W.; Tobias, D. J.; Mondragon-Ramirez, C.; Vorobyov, I.; MacKerell Jr, A. D.; Pastor, R. W. Update of the CHARMM All-atom Additive Force Field for Lipids: Validation on Six Lipid Types. *J. Phys. Chem. B* **2010**, *114*, 7830–7843, DOI: 10.1021/jp101759q.

(2) Lim, J. B.; Rogaski, B.; Klauda, J. B. Update of the Cholesterol Force Field Parameters in CHARMM. *J. Phys. Chem. B* **2012**, *116*, 203–210, DOI: 10.1021/jp207925m.

(3) Jorgensen, W. L.; Chandrasekhar, J.; Madura, J. D.; Impey, R. W.; Klein, M. L. Comparison of Simple Potential Functions for Simulating Liquid Water. *J. Chem. Phys.* **1983**, *79*, 926–935, DOI: 10.1063/1.445869.

(4) Lee, J.; Cheng, X.; Swails, J. M.; Yeom, M. S.; Eastman, P. K.; Lemkul, J. A.; Wei, S.; Buckner, J.; Jeong, J. C.; Qi, Y., et al. CHARMM-GUI Input Generator for NAMD, GROMACS, AMBER, OpenMM, and CHARMM/OpenMM Simulations Using the CHARMM36 Additive Force Field. *J. Chem. Theory Comput.* **2016**, *12*, 405–413, DOI: 10.1021/acs.jctc.5b00935.

(5) Abraham, M. J.; Murtola, T.; Schulz, R.; Páll, S.; Smith, J. C.; Hess, B.; Lindahl, E. GROMACS: High Performance Molecular Simulations through Multi-level Parallelism from Laptops to Supercomputers. *SoftwareX* **2015**, *1*, 19–25, DOI: 10.1016/j.softx.2015.06.001.

(6) Páll, S.; Zhmurov, A.; Bauer, P.; Abraham, M.; Lundborg, M.; Gray, A.; Hess, B.; Lindahl, E. Heterogeneous Parallelization and Acceleration of Molecular Dynamics Simulations in GROMACS. *J. Chem. Phys.* **2020**, *153*, 134110, DOI: 10.1063/5.0018516.

(7) Páll, S.; Hess, B. A Flexible Algorithm for Calculating Pair Interactions on SIMD Architectures. *Comput. Phys. Commun.* **2013**, *184*, 2641–2650, DOI: 10.1016/j.cpc.2013.06.003.

(8) Darden, T.; York, D.; Pedersen, L. Particle Mesh Ewald: An N·log(N) Method for Ewald Sums in Large Systems. *J. Chem. Phys.* **1993**, *98*, 10089–10092, DOI: 10.1063/1.464397.

(9) Essmann, U.; Perera, L.; Berkowitz, M. L.; Darden, T.; Lee, H.; Pedersen, L. G. A Smooth Particle Mesh Ewald Method. *J. Chem. Phys.* **1995**, *103*, 8577–8593, DOI: 10.1063/1.470117.

(10) Nosé, S. A Unified Formulation of the Constant Temperature Molecular Dynamics Methods. *J. Chem. Phys.* **1984**, *81*, 511–519, DOI: 10.1063/1.447334.

(11) Hoover, W. G. Canonical Dynamics: Equilibrium Phase-Space Distributions. *Phys. Rev. A* **1985**, *31*, 1695–1697, DOI: 10.1103/PhysRevA.31.1695.

(12) Parrinello, M.; Rahman, A. Polymorphic Transitions in Single Crystals: A New Molecular Dynamics Method. *J. Appl. Phys.* **1981**, *52*, 7182–7190, DOI: 10.1063/1.328693.

(13) Hess, B.; Bekker, H.; Berendsen, H. J.; Fraaije, J. G. LINCS: A Linear Constraint Solver for Molecular Simulations. *J. Comput. Chem.* **1997**, *18*, 1463–1472, DOI: 10.1002/(SICI)1096-987X(199709)18:12<1463::AID-JCC4>3.0.CO;2-H.

(14) Vögele, M.; Köfinger, J.; Hummer, G. Hydrodynamics of Diffusion in Lipid Membrane Simulations. *Phys. Rev. Lett.* **2018**, *120*, 268104.

(15) Ramasubramani, V.; Dice, B. D.; Harper, E. S.; Spellings, M. P.; Anderson, J. A.; Glotzer, S. C. freud: A Software Suite for High Throughput Analysis of Particle Simulation Data. *Comput. Phys. Commun.* **2020**, 107275, DOI: 10.1016/j.cpc.2020.107275.

(16) Virtanen, P. et al. SciPy 1.0: Fundamental Algorithms for Scientific Computing in Python. *Nat. Methods* **2020**, *17*, 261–272, DOI: 10.1038/s41592-019-0686-2.

(17) Ester, M.; Kriegel, H.-P.; Sander, J.; Xu, X., et al. A Density-based Algorithm for Discovering Clusters in Large Spatial Databases With Noise. Kdd. 1996; pp 226–231, DOI: 10.5555/3001460.3001507.

(18) Vermeer, L. S.; De Groot, B. L.; Réat, V.; Milon, A.; Czaplicki, J. Acyl Chain Order Parameter Profiles in Phospholipid Bilayers: Computation From Molecular Dynamics Simulations and Comparison With ^{2}H NMR Experiments. *Eur. Biophys. J.* **2007**, *36*, 919–931, DOI: 10.1007/s00249-007-0192-9.

(19) Lakowicz, J. R.; Bevan, D. R.; Maliwal, B. P.; Cherek, H.; Balter, A. Synthesis and characterization of a fluorescence probe of the transition and dynamic properties of membranes. *Biochemistry* **1983**, *22*, 5714–5722, DOI: 10.1021/bi00294a006.

(20) Scollo, F.; Evci, H.; Amaro, M.; Jurkiewicz, P.; Sykora, J.; Hof, M. What does time-dependent fluorescence shift (TDFS) in biomembranes (and proteins) report on? *Front. Chem.* **2021**, DOI: 10.3389/fchem.2021.738350.

(21) Jurkiewicz, P.; Olżyńska, A.; Langner, M.; Hof, M. Headgroup hydration and mobility of DOTAP/DOPC bilayers: a fluorescence solvent relaxation study. *Langmuir* **2006**, *22*, 8741–8749, DOI: 10.1021/la061597k.

(22) Kaiser, R. D.; London, E. Location of diphenylhexatriene (DPH) and its derivatives within membranes: comparison of different fluorescence quenching analyses of membrane depth. *Biochemistry* **1998**, *37*, 8180–8190, DOI: 10.1021/bi980064a.

(23) Franova, M.; Repakova, J.; Capkova, P.; Holopainen, J. M.; Vattulainen, I. Effects of DPH on DPPC- cholesterol membranes with varying concentrations of cholesterol: from local perturbations to limitations in fluorescence anisotropy experiments. *J. Phys. Chem. B* **2010**, *114*, 2704–2711, DOI: 10.1021/jp908533x.

(24) Parasassi, T.; De Stasio, G.; Ravagnan, G.; Rusch, R. M.; Gratton, E. Quantitation of

lipid phases in phospholipid vesicles by the generalized polarization of Laurdan fluorescence. *Biophys. J.* **1991**, *60*, 179–189, DOI: 10.1021/jp908533x.

(25) Lakowicz, J. R. *Principles of fluorescence spectroscopy*; Springer, 1983; pp 341–381.

(26) BIPM, I.; IFCC, I.; ISO, I.; IUPAP, O. JCGM 100: 2008 (GUM 1995 with minor corrections) Evaluation of measurement data–Guide to the expression of uncertainty in measurement. 2008.

(27) Harb, F.; Simon, A.; Tinland, B. Ripple Formation in Unilamellar-Supported Lipid Bilayer Revealed by FRAPP. *Eur. Phys. J. E* **2013**, *36*, 1–7, DOI: 10.1140/epje/i2013-13140-x.

(28) Wang, C.; Krause, M. R.; Regen, S. L. Push and Pull Forces in Lipid Raft Formation: The Push Can Be as Important as the Pull. *J. Am. Chem. Soc.* **2015**, *137*, 664–666, DOI: 10.1021/ja5115437.

(29) Gallová, J.; Uhríková, D.; Kučerka, N.; Doktorovová, S.; Funari, S. S.; Teixeira, J.; Balgavỳ, P. The Effects of Cholesterol and β-sitosterol on the Structure of Saturated Diacylphosphatidylcholine Bilayers. *Eur. Biophys. J.* **2011**, *40*, 153–163, DOI: 10.1007/s00249-010-0635-6.

(30) Léonard, A.; Escrive, C.; Laguerre, M.; Pebay-Peyroula, E.; Néri, W.; Pott, T.; Katsaras, J.; Dufourc, E. J. Location of Cholesterol in DMPC Membranes. A Comparative Study by Neutron Diffraction and Molecular Mechanics Simulation. *Langmuir* **2001**, *17*, 2019–2030, DOI: 10.1021/la001382p.
