# Supplementary material for: The Two Faces of the Liquid Ordered Phase
Source: J Phys Chem Lett. 2022 Feb 1;13(5):1307–13. doi: 10.1021/acs.jpclett.1c03712 (PMC8842317; doi:10.1021/acs.jpclett.1c03712)
Supplement: Supplementary file 2 — jz1c03712_si_002.pdf [file jz1c03712_si_002.pdf]

jz-2021-037123.R1

Name: Peer Review Information for "The Two Faces of the Liquid Ordered Phase"

First Round of Reviewer Comments

Reviewer: 1

Comments to the Author

Schachter et al performed molecular dynamics simulations, differential scanning calorimetry, and fluorescence spectroscopy to evaluate why molecules are often excluded from Lo phases. Overall, it is a carefully performed work. However, I have some major comments.

In this manuscript, the exclusion of molecules from Lo phase is attributed to the temperature-dependent bilayer structure in Lo phase. However, it is now well-appreciated in the field that exclusion of many of the “raft” molecules from Lo domains of synthetic vesicles is due to the extreme order of Lo and extreme disorder of Ld. That’s why, cell-derived vesicles are considered as better choice for partitioning experiments. This was shown several times by different labs (PMID: 25905447; PMID: 22450237; PMID: 19805351; PMID: 17360623; PMID: 17936718). Authors should discuss this thoroughly and how their data fits (or does not fit) into this concept.

In this study, authors used DPPC as a model for saturated lipid and most of the conclusions are based on the DPPC geometry. However, most of the ternary mixture data is based on sphingolipid mixtures (where the exclusion of molecules from Lo are often observed). Therefore, the exclusion of molecules from Lo phase cannot be specifically due to DPPC geometry. Instead, it should be a general property of Lo phase as in the point raised above. Authors should address this point.

II am not aware of any experimental evidence that a molecule partitions into Ld at room temperature and Lo at physiological temperature. If there is such studies, this would strengthen the claims in this manuscript. Also performing such a pilot experiment should not be difficult and authors can test their claims experimentally. Authors can pick a saturated lipid analog (that is supposed to partition in Lo but partitions in Ld) and test its partitioning in DPPC:DOPC:Chol mixture at room temperature and physiological temperature.

Minor

I understand the reason why authors show the temperature as Kelvin, but biology readers would appreciate more if temperature unit was Celsius. Also, authors should explain clearly why they picked these three temperatures.

Typo: gel phase to an o one.

Reviewer: 2

Comments to the Author

This contribution convincingly demonstrates that the famous liquid-ordered state of lipid membranes actually has two faces that differ in their local sub-structure. At temperatures below the main phase transition temperature (Tm) of the fully saturated component, small gel-type lipid clusters are observed, surrounded by more disordered regions enriched in cholesterol and fluid lipids. Above Tm, the Lo phase becomes homogeneous.

This, by itself, is an important finding. The results from MD simulations (on two different mixtures) are corroborated by a number of experimental assays, which adds to the credibility of this study. I am no expert on the experimental methods used, so cannot comment on their rigour, but the MD simulations appear solid and I have no reason to doubt these results, apart from some issues discussed below.

One major concern is the connection of the findings to the sorting of proteins in real membranes, in particular the raft-associated proteins. The authors do not explain clearly what kind of raft concept they have in mind (this is still an ill-defined concept), nor do they show any evidence that the 'high temperature' face of the Lo phase indeed facilitates the sorting of proteins into this phase. I suggest to move the part about protein sorting and rafts to the very end of the manuscript, as a more speculative paragraph discussing that the different faces of Lo domains could play a role in resolving discrepancies in the field, including the contradicting evidence of protein sorting in vivo and in vitro systems.

In connection to this point, rafts are typically associated with long tail SM lipids, which may have a Tm that is actually higher than DPPC and therefore one would expect that such rafts still contain the gel-type of domains as observed in the current paper - and therefore excluding proteins. Other evidence, also pointing to a less obvious explanation wrt protein sorting, is the fact that in coarse-grain simulations proteins tend to sort into disordered domains, despite the fact that the ordered domains in the CG model are resembling the high temperature Lo face. These issues deserve some proper discussion.

My second major concern has to do with hysteresis effects, always an issue when dealing with phase transitions. I realize that the current transitions seen between the two faces of Lo are not 1st order, but still it would be nice to see that the same final ensembles are obtained when starting from different ends of the temperature spectrum, i.e., starting from a high T face and simulate at low T, and vice versa. One could argue that the melting of the gel-clusters is kinetically faster for the ternary system due to the presence of the unsaturated lipids, giving rise to an apparent difference in the transition temperature between the binary and ternary mixtures.

Possible hysteresis is even more relevant for the pure DPPC simulation that is used as a reference in the SI - it appears only a cooling run was performed, but not a heating run. In addition, pinpointing a transition temperature for pure DPPC is non-trivial for a number of other reasons: (i) DPPC actually forms a rippled phase in between of the gel and fluid phases, (ii) the transition temperature is very sensitive to hydration levels - in fact the hydration level changes during the real transition, which is difficult to capture in a simulation, and (iii) for ideal packing of the tails an anisotropic box shape is required. These issues should be discussed, and the apparent Tm obtained for pure DPPC should be put into the proper perspective.

Given the above considerations, I am not entirely convinced that the force field that is being used is actually capturing the Tm of DPPC accurately. Suppose that the force field actually overestimates Tm, i.e., favouring the gel phase. This could imply that the binary and ternary systems at lower temperatures would like to form a coexisting gel-Lo phase, which may be hindered by the limited sample size and simulation time, leading to a meta-stable phase characterized by small gel clusters. If the authors could perform an additional simulation, starting from such a potential gel-liquid biphasic system, and see the break up of the gel domain into small gel clusters, I would be much more convinced that we are not looking at an artefact of the force field model.

Although I appreciate the large amount of analysis performed on the simulated systems, I was missing an analysis to quantify the lateral homogeneity in the systems, such as preferential solvation. This would be a direct measure of the appearance/disappearance of the DPPC gel clusters with temperature, and also inform us about possible residual inhomogeneities of the high T Lo face.

A final remark concerns the preference for cholesterol to be closest to the 'free' lipid tails. If I remember the work from Lyman et al. well, I recall they observe a strong clustering of cholesterol around the edges of the gel-domains. Is there a real difference here, or more a matter of analysis details ?

Reviewer: 3

Comments to the Author

The manuscript by Javanainen and coworkers presents interesting and new results regarding the structure and thermodynamics of the liquid ordered (Lo) phase of lipid bilayers. The Lo phase has long fascinated the field of membrane biology and biophysics as a possible model for the organization of living cell membranes, but has also fallen short in a number of respects. In particular, the partitioning of membrane proteins between different kinds of coexisting phases in model Lo systems has often not been faithful to results obtained for more realistic systems. Part of the issue in the field has been a proliferation of results and interpretations, leading to confusion. Although this may appear to be somewhat specialized, it should be noted that there is an enormous literature on this topic, stretching back more than 20 years, and it is rife with confusion and inconsistencies. The present manuscript presents significant and new results which ought to help resolve some of this confusion, and thus I expect will have a significant impact. In addition the combination of careful simulations and experimental measurements ought to serve as a roadmap to researchers in the rapidly growing field of liquid-liquid "phase separation" in cells, which would benefit from a more rigorous understanding of phase transitions.

The present submission presents very thorough and comprehensive results obtained by three different experimental techniques and by all-atom simulations, which together provide new insight into the molecular and thermodynamic details of the Lo phase. The authors show quite convincingly that the hexagonal substructure within the Lo phase (first reported by Sodt et al in 2014) melts with increasing temperature, which they call Tco. Below this temperature is observed the substructure reported buy Sodt et al, and above this temperature there is no longer any substructure, but cholesterol still has an ordering effect on nearby chains. Importantly, some commonly used experimental measurements cannot distinguish between these two cases, which likely has led to considerable confusion in the field.

I have only a few minor suggestions, mostly typos and editorial comments:

(1) It might enhance the interest of the submission to discuss in more detail the relation between the "traditional" Lo phases of binary and ternary mixtures studied here, and the ordered phases observed in more complex (eg GPMV) mixtures. Levental and coworkers have extensively reported on this, and shown that in more complex mixtures the standard observables are more similar to one another in the Lo and Ld phases than in ternary mixtures, where they are more extreme. What might this mean in the context of the new results reported here? And, do the authors expect that cell membranes at physiological temperatures are like the Lo phase above Tco? (The later seems a reasonable suggestion to me.)

(2) In the abstract, it is stated that "raft-associated proteins exclusively partition to the Ld and not Lo phase in these model systems” – I think you mean multi pass membrane proteins. Lipid anchored

proteins and single pass TMs certainly can partition to Lo. See eg LAT, palmitoyl anchored, G-proteins, etc.

(3) Introduction: “tiny ordered lipid domains” can you be more quantitative? What is tiny?

(4) Intro: “break the gel phase to an o one” typo.

(5) Intro: “DPPC chain conformations change from all-anti at 293 K to gauche

at 333 K.” probably not quite accurate…mostly trans/anti to a larger fraction of gauche

(6) figs are out of order. Fig 2 discussed, then fig 4.

(7) How is chain orientation defined, and what does “tilt” for a single chain mean, esp at higher T where the chains are disordered?

Author's Response to Peer Review Comments:

Reviewer: 1

Reviewer comment:
Schachter et al performed molecular dynamics simulations, differential scanning calorimetry, and fluorescence spectroscopy to evaluate why molecules are often excluded from Lo phases. Overall, it is a carefully performed work. However, I have some major comments.

In this manuscript, the exclusion of molecules from Lo phase is attributed to the temperature-dependent bilayer structure in Lo phase. However, it is now well-appreciated in the field that exclusion of many of the "raft" molecules from Lo domains of synthetic vesicles is due to the extreme order of Lo and extreme disorder of Ld. That's why, cell-derived vesicles are considered as better choice for partitioning experiments. This was shown several times by different labs (PMID: 25905447; PMID: 22450237; PMID: 19805351; PMID: 17360623; PMID: 17936718). Authors should discuss this thoroughly and how their data fits (or does not fit) into this concept.

Author Reply:
This is an excellent point, and GPMVs indeed provide a more faithful system to study protein partitioning than synthetic vesicles do due to the higher ordering of the L_o phase in synthetic systems. We believe that our present manuscript provides an explanation for this "extreme order". Nevertheless, even GPMVs only phase-separate at low temperatures (well below the physiological temperature), indicating that the structure of the L_o phase even in these model systems might not correspond to the structure of ordered domains in the plasma membrane. Unfortunately, no phase diagrams let alone tie lines are available for the complex lipid mixtures present in GPMVs, preventing us from simulating the L_o phase of phase-separated GPMVs to study its molecular structure. Experimental studies using GPMVs also pose several technical difficulties and give less control over their composition as compared to synthetic vesicles.

We have cited all the papers pointed out by the Reviewer in the revised manuscript. We have extended the discussion on the similarities and differences between synthetic vesicles, GPMVs, and *in vivo* plasma membranes in the revised manuscript. Notably, we now hypothesise that in GPMVs and the plasma membrane, the two distinct molecular structures observed for the L_o phase in different temperature regimes might coexist, and their presence could depend on the local critical fluctuations in both composition and temperature. We agree that the application of GPMVs can be beneficial in our future studies.

Reviewer Comment:
In this study, authors used DPPC as a model for saturated lipid and most of the conclusions are based on the DPPC geometry. However, most of the ternary mixture data is based on sphingolipid mixtures (where the exclusion of molecules from Lo are often observed). Therefore, the exclusion of molecules from Lo phase cannot be specifically due to DPPC geometry. Instead, it should be a general property of Lo phase as in the point raised above. Authors should address this point.

Author Reply:
We never intended to highlight DPPC geometry as the driving factor behind the observed behavior. Instead, we would expect sphingolipids to behave in a similar manner since they also form hexagonally-packed and cholesterol-depleted regions at lower temperatures (DOI: 10.1016/j.bpj.2015.07.036) that we believe to be behind the exclusion of many raft-associated molecules from this phase.

For this study, we chose the mixtures of DPPC/DOPC/cholesterol and DPPC/cholesterol since they are the most studied binary and ternary lipid mixtures associated with the L_o phase. Sphingolipids bring with them specific hydrogen bonding patterns, and are a natural extension to our work. Indeed, We aim to clarify the temperature-dependence of mixtures containing sphingomyelin with our ongoing simulations of the PSM/DOPC/CHOL mixtures displaying coexistence.

We have added a sentence in the discussion highlighting the similarity of the molecular structure in mixtures containing saturated phospholipids and sphingolipids.

Reviewer Comment:
I am not aware of any experimental evidence that a molecule partitions into Ld at room temperature and Lo at physiological temperature. If there is such studies, this would strengthen the claims in this manuscript. Also performing such a pilot experiment should not be difficult and authors can test their claims experimentally. Authors can pick a saturated lipid analog (that is supposed to partition in Lo but partitions in Ld) and test its partitioning in DPPC:DOPC:Chol mixture at room temperature and physiological temperature.

Author Reply:
The main issue here is that neither synthetic mixtures of, *e.g.*, DPPC/DOPC/CHOL nor GPMVs sustain L_o/L_d phase separation at the physiological temperature. In our manuscript, we tried to point out this discrepancy, as we believe that any measurements performed at room temperature or below might not mimic cellular conditions at a physiological temperature. Still, many proteins are considered to be raft-associated based on different biochemical assays performed *in vivo*, although they partition to the disordered phase in synthetic vesicles. Unfortunately, direct measurements on raft affinity *in vivo* are unfeasible, and cannot be performed to clarify this issue. We have expanded our discussion on protein partitioning in the

revised manuscript. This discussion is located at the very end of the manuscript, since it is often speculative in nature, and since it is only a possible implication of the actual results of the paper regarding the two molecular structures of the L_o phase.

Reviewer Comment:
I understand the reason why authors show the temperature as Kelvin, but biology readers would appreciate more if temperature unit was Celsius. Also, authors should explain clearly why they picked these three temperatures.

Author Reply:
We understand the reviewer's point. However, some readers in the field of computational biophysics will certainly appreciate the usage of Kelvin more. Moreover, as the ACS style guide says *"Where possible, use metric and SI units",* we have decided to provide temperatures in Kelvin also in the revised manuscript.

Regarding the use of temperatures, both simulations and fluorescence experiments were performed at many more than three different temperatures. Similarly, the DSC measurements span a similar range of temperatures. The used temperatures cover a range that is of interest in biophysics and physical chemistry, namely from slightly below room temperature to above the body temperature, and the crossover temperatures of the used mixtures fortunately fall within this range.

Reviewer Comment:
Typo: gel phase to an o one.

Author Reply:
We have corrected this mistake, and the text now reads "*gel phase to an L_o one"*.

Reviewer: 2

Reviewer Comment:
This contribution convincingly demonstrates that the famous liquid-ordered state of lipid membranes actually has two faces that differ in their local sub-structure. At temperatures below the main phase transition temperature (Tm) of the fully saturated component, small gel-type lipid clusters are observed, surrounded by more disordered regions enriched in cholesterol and fluid lipids. Above Tm, the Lo phase becomes homogeneous.

This, by itself, is an important finding. The results from MD simulations (on two different mixtures) are corroborated by a number of experimental assays, which adds to the credibility of this study. I am no expert on the experimental methods used, so cannot comment on their rigour, but the MD simulations appear solid and I have no reason to doubt these results, apart from some issues discussed below.

Author Reply:
We thank the Reviewer for the positive feedback.

Reviewer Comment:
One major concern is the connection of the findings to the sorting of proteins in real membranes, in particular the raft-associated proteins. The authors do not explain clearly what kind of raft concept they have in mind (this is still an ill-defined concept), nor do they show any evidence that the 'high temperature' face of the Lo phase indeed facilitates the sorting of proteins into this phase. I suggest to move the part about protein sorting and rafts to the very end of the manuscript, as a more speculative paragraph discussing that the different faces of Lo domains could play a role in resolving discrepancies in the field, including the contradicting evidence of protein sorting in vivo and in vitro systems.

Author Reply:
We agree with the Reviewer that the raft concept is ill-defined, and the related literature often uses "*raft*", *"ordered phase"*, and *"domain"* almost interchangeably, creating even more confusion in the field. We believe that the putative rafts in the plasma membrane could be a manifestation of the high-temperature L_o phase, explaining the association of many proteins with rafts despite their L_d partitioning preference in synthetic vesicles and even GPMVs. Still, due to this ambiguity and following the Reviewer's advice, we have extended the discussion covering protein partitioning at the end of the revised manuscript, and changed the tone of our writing into a more cautious one.

Reviewer Comment:
In connection to this point, rafts are typically associated with long tail SM lipids, which may have a Tm that is actually higher than DPPC and therefore one would expect that such rafts still contain the gel-type of domains as observed in the current paper - and therefore excluding proteins. Other evidence, also pointing to a less obvious explanation wrt protein sorting, is the fact that in coarse-grain simulations proteins tend to sort into disordered domains, despite the fact that the ordered domains in the CG model are resembling the high temperature Lo face. These issues deserve some proper discussion.

Author Reply:
The Reviewer makes an excellent and helpful point regarding the long tail SM lipids. We have included this point to the improved discussion section at the end of the revised manuscript. Notably, we highlight the possibility that fluctuations in temperature and composition lead to the assembly of L_o-like domains that can represent the two molecular structures observed for the L_o phase based on their local composition.

We believe that the Reviewer refers to coarse-grained simulations based on the Martini model. However, we strongly disagree that the ordered phase observed in Martini simulations would correspond to the high-temperature L_o phase observed in the present manuscript: We have very recently demonstrated that the constrained structure of Martini cholesterol, combined with the use of relatively large time steps common for Martini simulations, leads to artificial temperature gradients in simulations of phase-separated systems (DOI:10.1021/acs.jpcb.1c03665). Therefore, the temperature of the ordered phase in these simulations can be dozens of degrees lower than the target temperature of the thermostat, leading to very sharp phase boundaries and a very stiff ordered phase. We believe that this artefact haunts most—if not all—of the phase coexistence studies performed using the Martini model. In this light, it is not surprising that no proteins have partitioned to this ordered phase. Our preliminary simulations with the parameters that prevent the emergence of artificial temperature gradients display significantly more fluid phase boundaries, and a more significant partitioning of saturated chains to the disordered phase and unsaturated chains to the ordered phase. Therefore, future coarse-grained simulations will certainly have the power to clarify the partitioning preferences of membrane proteins, and the results will likely be different from those available in the present literature.

Reviewer Comment:
My second major concern has to do with hysteresis effects, always an issue when dealing with phase transitions. I realize that the current transitions seen between the two faces of Lo are not 1st order, but still it would be nice to see that the same final ensembles are obtained when starting from different ends of the temperature spectrum, i.e., starting from a high T face and simulate at low T, and vice versa. One

could argue that the melting of the gel-clusters is kinetically faster for the ternary system due to the presence of the unsaturated lipids, giving rise to an apparent difference in the transition temperature between the binary and ternary mixtures.

Author Reply:
This is a great suggestion for a sanity check. We have now performed two additional simulations with the ternary mixture:

1. At 293 K, starting with the final structure of the original simulation at 333 K
2. At 333 K, starting with the final structure of the original simulation at 293 K

We have analyzed some central properties from these simulations following the same protocols as used for the initial set of simulations. These results are included in Figs. 2 & 3 as black crosses. Importantly, these new results are essentially identical to the initial data set, indicating that hysteresis does not play a role in the observed behavior, at least in the probed microsecond time scale.

In the revised manuscript, we mention these additional simulations in the methods section, in Table S1, and in the captions of Figs. 2 & 3. We also point out that they provide essentially identical results to the original simulation set, indicating that our observations are free of hysteresis effects.

Reviewer Comment:
Possible hysteresis is even more relevant for the pure DPPC simulation that is used as a reference in the SI - it appears only a cooling run was performed, but not a heating run. In addition, pinpointing a transition temperature for pure DPPC is non-trivial for a number of other reasons: (i) DPPC actually forms a rippled phase in between of the gel and fluid phases, (ii) the transition temperature is very sensitive to hydration levels - in fact the hydration level changes during the real transition, which is difficult to capture in a simulation, and (iii) for ideal packing of the tails an anisotropic box shape is required. These issues should be discussed, and the apparent Tm obtained for pure DPPC should be put into the proper perspective.

Author Reply:
This is an excellent point. In our further tests the hysteresis indeed caused problems, and we have thus decided to remove the annealing simulation from the manuscript, since they were not even referred to in the main text.

Reviewer Comment:
Given the above considerations, I am not entirely convinced that the force field that is being used is actually capturing the Tm of DPPC accurately. Suppose that the force field actually overestimates Tm, i.e., favouring the gel phase. This could imply that the binary and ternary systems at lower temperatures would like to form a coexisting gel-Lo phase, which may be hindered by the limited sample size and simulation time, leading to a meta-stable phase characterized by small gel clusters. If the authors could perform an additional simulation, starting from such a potential

gel-liquid biphasic system, and see the break up of the gel domain into small gel clusters, I would be much more convinced that we are not looking at an artefact of the force field model.

Author Reply:
The lipid mixtures used in this work contain 30% cholesterol, which places them well into the L_o regions of the respective phase diagrams. For example in the binary system, a gel/L_o coexistence would be observed below T_m at a CHOL concentration range between ~10% and ~20% (DOI: 10.1021/bi00454a021).

Thus, we believe that the only thing that could be affected by an imperfect T_m in the force field would be the crossover temperature T_{co}. Simulations by other groups utilizing the same force field place the T_m of DPPC either at 317 K (DOI: 10.1101/2021.11.25.470048) or between 308 and 313 K (DOI: 10.1016/j.bbamem.2018.04.014), suggesting that it does not differ significantly from the experimental estimate of 314 K. Such a small deviation does not affect our conclusion that the L_o phase covers two different molecular structures, they are separated by a subtle transition, and this transition takes place at a temperature that falls in the range from room to physiological temperature. Furthermore, the consistency of the used model is further supported by the good agreement between the T_{co} values extracted from our simulations and experiments.

Reviewer Comment:
Although I appreciate the large amount of analysis performed on the simulated systems, I was missing an analysis to quantify the lateral homogeneity in the systems, such as preferential solvation. This would be a direct measure of the appearance/disappearance of the DPPC gel clusters with temperature, and also inform us about possible residual inhomogeneities of the high T Lo face.

Author Reply:
This is a good point. Such an analysis was actually performed, and the results are provided in the caption of Fig. S6 in the Supplementary Information. There is some preferential interaction between DPPC and CHOL even at higher temperatures, in line with experimental studies (DOI: 10.1021/ja5115437).

Reviewer Comment:
A final remark concerns the preference for cholesterol to be closest to the 'free' lipid tails. If I remember the work from Lyman et al. well, I recall they observe a strong clustering of cholesterol around the edges of the gel-domains. Is there a real difference here, or more a matter of analysis details ?

Author Reply:
This is certainly the same effect, which is pointed out more clearly in the revised manuscript.

Reviewer: 3

Reviewer Comment:

The manuscript by Javanainen and coworkers presents interesting and new results regarding the structure and thermodynamics of the liquid ordered (Lo) phase of lipid bilayers. The Lo phase has long fascinated the field of membrane biology and biophysics as a possible model for the organization of living cell membranes, but has also fallen short in a number of respects. In particular, the partitioning of membrane proteins between different kinds of coexisting phases in model Lo systems has often not been faithful to results obtained for more realistic systems. Part of the issue in the field has been a proliferation of results and interpretations, leading to confusion. Although this may appear to be somewhat specialized, it should be noted that there is an enormous literature on this topic, stretching back more than 20 years, and it is rife with confusion and inconsistencies. The present manuscript presents significant and new results which ought to help resolve some of this confusion, and thus I expect will have a significant impact. In addition the combination of careful simulations and experimental measurements ought to serve as a roadmap to researchers in the rapidly growing field of liquid-liquid "phase separation" in cells, which would benefit from a more rigorous understanding of phase transitions.

The present submission presents very thorough and comprehensive results obtained by three different experimental techniques and by all-atom simulations, which together provide new insight into the molecular and thermodynamic details of the Lo phase. The authors show quite convincingly that the hexagonal substructure within the Lo phase (first reported by Sodt et al in 2014) melts with increasing temperature, which they call Tco. Below this temperature is observed the substructure reported buy Sodt et al, and above this temperature there is no longer any substructure, but cholesterol still has an ordering effect on nearby chains. Importantly, some commonly used experimental measurements cannot distinguish between these two cases, which likely has led to considerable confusion in the field.

Author Reply:

We thank the reviewer for the kind words.

I have only a few minor suggestions, mostly typos and editorial comments:

Reviewer Comment:

(1) It might enhance the interest of the submission to discuss in more detail the relation between the "traditional" Lo phases of binary and ternary mixtures studied here, and the ordered phases observed in more complex (eg GPMV) mixtures. Levental and coworkers have extensively reported on this, and shown that in more complex mixtures the standard observables are more similar to one another in the Lo

and Ld phases than in ternary mixtures, where they are more extreme. What might this mean in the context of the new results reported here? And, do the authors expect that cell membranes at physiological temperatures are like the Lo phase above Tco? (The later seems a reasonable suggestion to me.)

Author Reply:
This is an excellent point. Indeed, the two phases in GPMVs are more similar than those in synthetic mixtures, and we now discuss property in our revised manuscript. Still, even GPMVs only phase-separate at temperatures well below the body temperature. Thus, in the case that the plasma membrane contained small ordered domains or "rafts", it is likely that these domains would result from critical fluctuations rather than thermodynamic phase separation. However, the plasma membrane is extremely complex in composition and contains some sphingolipids with very high melting points. Therefore, it is possible that the compositions of the ordered domains in the plasma membrane differ significantly, possibly manifesting both of the faces of the L_o phase we report in our manuscript. We have now included this possibility in the discussion at the end of our revised manuscript.

Reviewer Comment:
(2) In the abstract, it is stated that "raft-associated proteins exclusively partition to the Ld and not Lo phase in these model systems" – I think you mean multi pass membrane proteins. Lipid anchored proteins and single pass TMs certainly can partition to Lo. See eg LAT, palmitoyl anchored, G-proteins, etc.

Author Reply:
Unfortunately, we are unaware of any single-pass TMs that would partition to the L_o phase in synthetic model membranes. However, this is indeed true for some proteins in GPMVs. Moreover, a recent study observed a multi-pass membrane protein to partition to the ordered phase in GPMVs, although it partitioned to the disordered phase in synthetic vesicles (DOI:10.1073/pnas.2000508117). We have clarified this point, and included the new references to point out the differences between GPMVs and synthetic systems and highlight protein partitioning in GPMVs.

Reviewer Comment:
(3) Introduction: "tiny ordered lipid domains" can you be more quantitative? What is tiny?

Author Reply:
This is a good point. We have replaced "tiny" by "nanoscale". Still, we call these rafts "putative" in the sentence and thus want to avoid speculating on their size.

Reviewer Comment:
(4) Intro: “break the gel phase to an o one” typo.

Author Reply:
We have corrected this mistake, and the text now reads “*gel phase to an L_o one*”.

Reviewer Comment:
(5) Intro: “DPPC chain conformations change from all-anti at 293 K to gauche at 333 K.” probably not quite accurate…mostly trans/anti to a larger fraction of gauche

Author Reply:
We have corrected this expression as suggested by the reviewer in the revised manuscript.

Reviewer Comment:
(6) figs are out of order. Fig 2 discussed, then fig 4.

Author Reply:
This is a conscious choice, as all experiments are grouped in one figure (Fig. 4) to be close to discussion related to the fluorescence results. However, DSC results (Fig. 4A) are briefly compared to simulations earlier in the text, leading to this 2–4–3 ordering of the Figure references. Still, we believe that having the experimental results in one figure close to where they are discussed is more important than having the references be consecutively numbered. If our manuscript gets accepted, we will return to this issue with the ACS production team.

Reviewer Comment:
(7) How is chain orientation defined, and what does “tilt” for a single chain mean, esp at higher T where the chains are disordered?

Author Reply:
This is a good point. Indeed, at higher temperatures the tilt can be somewhat misleading since the chains no longer behave as rigid objects. At these higher temperatures, the deuterium order parameters—which are also analyzed in our manuscript—provide more information on the chain conformations. However, we have analyzed the chain tilting, since at low temperatures this parameter can distinguish between the gel phase (tilt angle of ~25 degrees) and the lipid ordered phase (no common tilt angle), although their order parameter values can be very similar.

The methodology for calculating lipid chain tilt is defined in Section S3 in the Supplementary Information.
